# A Review on Systems-Based Sensory Gloves for Sign Language Recognition State of the Art between 2007 and 2017

**DOI:** 10.3390/s18072208

**Published:** 2018-07-09

**Authors:** Mohamed Aktham Ahmed, Bilal Bahaa Zaidan, Aws Alaa Zaidan, Mahmood Maher Salih, Muhammad Modi bin Lakulu

**Affiliations:** 1 Department of Computing, Universiti Pendidikan Sultan Idris, Tanjong Malim, Perak 35900, Malaysia; mohamed.aktham3@gmail.com (M.A.A.); bilalbahaa@fskik.upsi.edu.my (B.B.Z.); mahmaher1989@gmail.com (M.M.S.); modi@fskik.upsi.edu.my (M.M.B.L.); 2 Department of Computer Science, Computer Science and Mathematics College, Tikrit University, Tikrit 34001, Iraq

**Keywords:** sign language, glove, sensor, gesture recognition, pattern recognition, man-machine interface (MMI), classification

## Abstract

Loss of the ability to speak or hear exerts psychological and social impacts on the affected persons due to the lack of proper communication. Multiple and systematic scholarly interventions that vary according to context have been implemented to overcome disability-related difficulties. Sign language recognition (SLR) systems based on sensory gloves are significant innovations that aim to procure data on the shape or movement of the human hand. Innovative technology for this matter is mainly restricted and dispersed. The available trends and gaps should be explored in this research approach to provide valuable insights into technological environments. Thus, a review is conducted to create a coherent taxonomy to describe the latest research divided into four main categories: development, framework, other hand gesture recognition, and reviews and surveys. Then, we conduct analyses of the glove systems for SLR device characteristics, develop a roadmap for technology evolution, discuss its limitations, and provide valuable insights into technological environments. This will help researchers to understand the current options and gaps in this area, thus contributing to this line of research.

## 1. Introduction

According to the statistics of the World Federation of the Deaf and the World Health Organization, approximately 70 million people in the world are deaf–mute. A total of 360 million people are deaf, and 32 million of these individuals are children [1]. The majority of speech- and hearing-impaired people cannot read or write in regular languages. Sign language (SL) is the native language used by the deaf and mute to communicate with others. SL relies primarily on gestures rather than voice to convey meaning, and it combines the use of finger shapes, hand movements, and facial expressions [2]. This language has the following main defects: a lot of hand movements, a limited vocabulary, and learning difficulties. Furthermore, SL is unfamiliar to those who are not deaf and mute, and disabled people face serious difficulties in communicating with able individuals. This communication barrier adversely affects the lives and social relationships of deaf people [3]. Thus, dumb people need to use a translator device to communicate with able individuals. This can be achieved by developing a glove equipped with sensors and an electronic circuit. Several benefits of using this device are that no complex data processing is needed [4]; there are no limitations on movements such as sitting behind a desk or chair; hand shape recognition is not affected by the background condition [5,6]; it is a lightweight, SLR-based glove device that can be carried to make mobility easy and comfortable [7,8]; and it is a recognition system that can be employed for learning SL for both dumb and able people [9]. Furthermore, numerous kinds of applications are currently involved in gesture recognition systems such as SLR, substitutional computer interfaces, socially assistive robotics, immersive gaming, virtual objects, remote control, medicine-health care, gesture recognition of hand/body language, etc. [10,11,12,13,14,15]. These benefits are described in detail in Section 5.1. The goal of this study is to survey glove systems used for SLR to obtain knowledge on current systems in this field. In addition, a roadmap is presented for technology evolution; it exhibits features of SLR systems and discusses the limitations of current technology. This objective can be achieved by answering the following six research questions: (1) How many types of relevant studies are published on glove-based SLR? (2) To what extent have SLR systems been elaborated on in the preliminary studies in terms of detail and technologies? (3) Which types of movements have been recognized in previous studies? (4) What types of sensors have been used in the primary publications? (5) What languages did the glove systems develop for SLR in the preliminary studies? (6) How were the effectiveness and efficiency of the SLR systems evaluated in the initial studies? The aim was to understand the options and gaps available in this field to support researchers by providing valuable insights into technological environments. The steps taken in this study to provide Systematic Literature Review and Systematic Mapping Study are in accordance with the steps explained in the David Moher paper [16] and other research papers [11,17,18,19,20,21].

**Figure 1:**
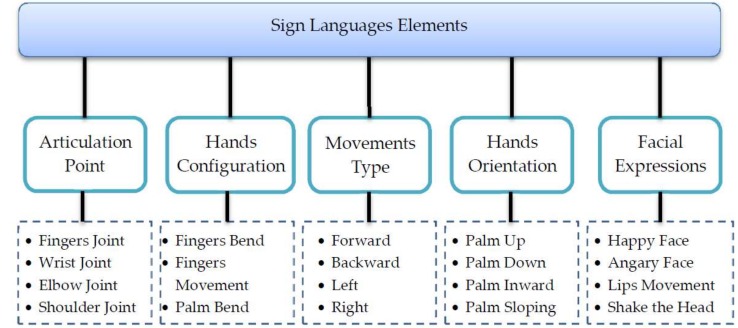
The essential elements related to sign language gesture formation.

SL is a visual–spatial language based on positional and visual components, such as the shape of fingers and hands, the location and orientation of the hands, and arm and body movements. These components are used together to convey the meaning of an idea. The phonological structure of SL generally has five elements (Figure 1). Each gesture in SL is a combination of five building blocks. These five blocks represent the valuable elements of SL and can be exploited by automated intelligent systems for SL recognition (SLR) [22].

**Figure 2:**
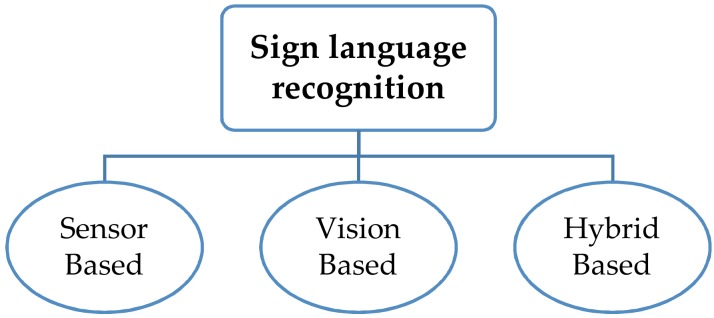
Sign language recognition approaches.

Scholarly interventions to overcome disability-related difficulties are multiple and systematic and vary according to the context. One of the important interventions is SLR systems that are utilized to translate the signs of SL into text or speech to establish communication with individuals who do not know these signs [18]. SLR systems based on the sensory glove are among the most significant endeavors aimed at procuring data for the motion of human hands. Three approaches (Figure 2), namely, vision based, sensor-based, and a combination of the two, are adopted to capture hand configurations and recognize the corresponding meanings of gestures [23].

**Figure 3:**
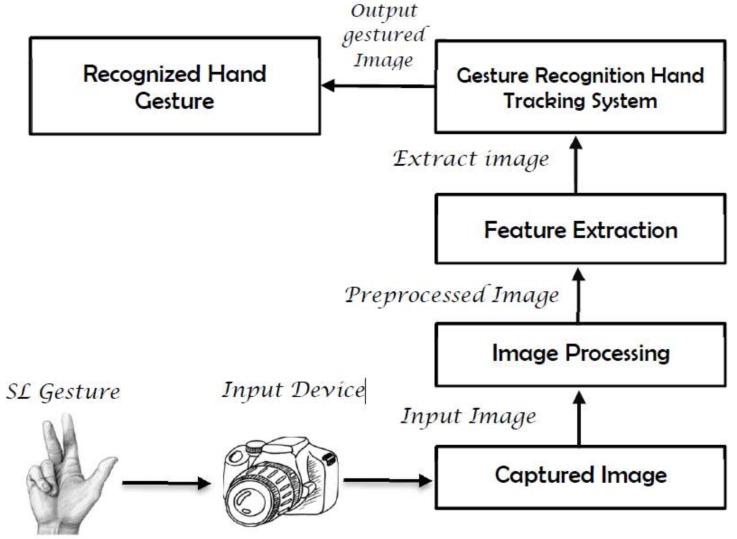
A flow chart of the processing steps used in the vision-based system for SLR.

Vision-based systems use cameras as primary tools to obtain the necessary input data (Figure 3). The main advantage of using a camera is that it removes the need for sensors in sensory gloves and reduces the building costs of the system. Cameras are quite cheap, and most laptops use a high specification camera because of the blur caused by a web camera. However, despite the high specification camera, which most smartphones possess [18], there are various problems such as the limited field of view of the capturing device, high computational costs [24,25], and the need for multiple cameras to obtain robust results (due to problems of depth and occlusion [26,27]); these issues are inherent to this system and render the entire system futile for the development of real-time recognition applications. In [28], two new feature extraction techniques of Combined Orientation Histogram and Statistical (COHST) Features and Wavelet Features are presented for the recognition of static signs of numbers 0 to 9, of American Sign Language (ASL). System performance is measured by extracting four different features—Orientation Histogram, Statistical Measures, COHST Features, and Wavelet Features—using a neural network. The best performance of the system reaches 98.17% accuracy with Wavelet features. In [29], a system using Wavelet transform and neural networks (NN) is presented to recognize the static gestures of alphabets in Persian sign language (PSL). It is able to recognize 32 selected PSL alphabets with an average recognition rate of 94.06%. In [30], ASL recognition is performed using Hough transform and neural NN. Here, only 20 different signs of alphabets and numbers were used. The performance of the system was measured by varying the threshold level for Canny edge detection and the number of samples for each sign used. The average recognition rate obtained was 92.3% for the threshold value of 0.25. In [31], a vision-based system with B-Spline Approximation and a support vector machines (SVM) classifier were used for the recognition of Indian Sign Language Alphabets and Numerals. Fifty samples of each alphabet from A–Z and numbers from 0–5 were used, and the system achieved an average accuracy of 90% and 92% for alphabets and numbers, respectively. In [32], a 3D model of the hand posture is generated from two 2D images from two perspectives that are weighted and linearly combined to produce single 3D features aimed at classifying 50 isolated ArsL words using a Hybrid pulse-coupled neural network (PCNN) as the feature generator technique, followed by nondeterministic finite automaton (NFA). Then, the ‘‘best-match” algorithm is used to find the most probable meaning of a gesture. The recognition accuracy reaches 96%. In [33], a combination of local binary patterns (LBP) and principal component analysis (PCA) is used to extract features that are fed into a Hidden Markov Model (HMM) to recognize a lexicon of 23 isolated ArSL words. Occlusion is not resolved, as any occlusion states are handled as one object, and recognition is carried out. The system achieves a recognition rate of 99.97% in signer-dependent mode. A dynamic skin detector based on the face color tone is used in [34] for hand segmentation. Then, a skin-blob tracking technique is used to identify and track the hands. A dataset of 30 isolated words is used. The proposed system has a recognition rate of 97%. Different transformation techniques (viz. Fourier, Hartley, and Log-Gabor transforms) were used in [27], for the extraction and description of features from an accumulation of sign frames into a single image of an Arabic Sign language dataset. Three transformation techniques were applied in total, as well as slices from an accumulation of sign frames. The system was tested using three classifiers: k-nearest neighbor (KNN), SVM, and. Overall, the system’s accuracy reached over 98% for Hartley transform, which is comparable with other works using the same dataset.

**Figure 4:**
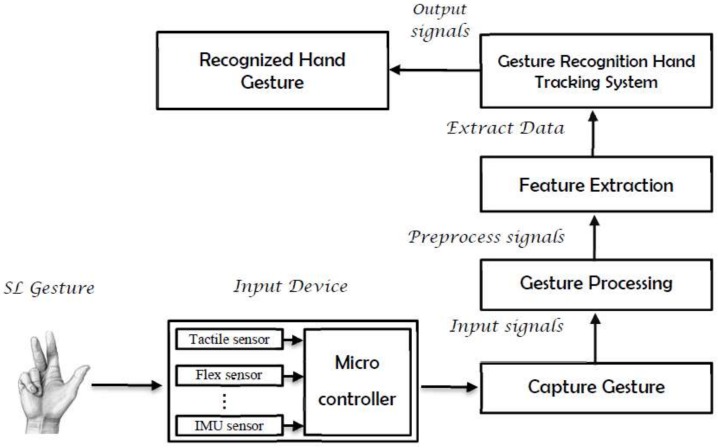
The main phases with regard to collecting and recognizing SL gestures data using the glove-based system.

The use of a certain type of instrumented gloves that are fitted with various sensors, namely, flexion (or bend) sensors, accelerometers (ACCs), proximity sensors, and abduction sensors, is an alternative approach with which to acquire gesture-related data (Figure 4). These sensors are used to measure the bend angles for fingers, the abduction between fingers, and the orientation (roll, pitch, and yaw) of the wrist. Degrees of freedom (DoF) that can be realized using such gloves vary from 5 to 22, depending on the number of sensors embedded in the glove. A major advantage of glove-based systems over vision-based systems is that gloves can directly report relevant and required data (degree of bend, pitch, etc.) in terms of voltage values to the computing device [35], thus eliminating the need to process raw data into meaningful values. By contrast, vision-based systems need to apply specific tracking and feature extraction algorithms to raw video streams, thereby increasing the computational overhead [10,36]. Later, we will review the articles related to this approach in detail.

The third method of collecting raw gesture data employs a hybrid approach that combines glove- and camera-based systems. This approach uses mutual error elimination to enhance the overall accuracy and precision. However, not much work has been carried out in this direction due to the cost and computational overheads of the entire setup. Nevertheless, augmented reality systems produce promising results when used with hybrid tracking methodology [3].

## 2. Materials and Methods

Painstaking and intensive endeavors to find realistic and feasible solutions to overcome communication obstacles are major challenges encountered by the deaf and mute. Therefore, the implementation of a system that identifies SL in its software and hardware branches has been emphasized.

### 2.1. Pertaining to System Materials

**Figure 5:**
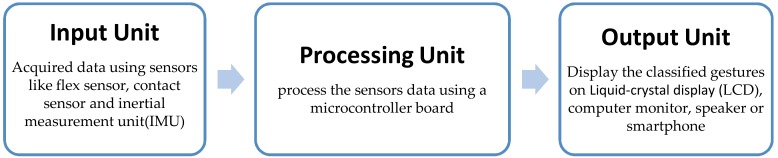
The main hardware components of the glove-based system.

With regard to hardware components, the glove-based recognition system is composed of three main units (Figure 5): input, processing, and output.

#### 2.1.1. Input Unit

Owing to scientific and technical developments in the field of electronic circuits, sensors have attracted attention, which have resulted in investments in sensor features in many small and large applications. The sensor is the main player in measuring hand data in terms of bending (shape), movement, rotation, and position of the hand.

a. Sensors Used to Detect Finger Bending

**Figure 6:**
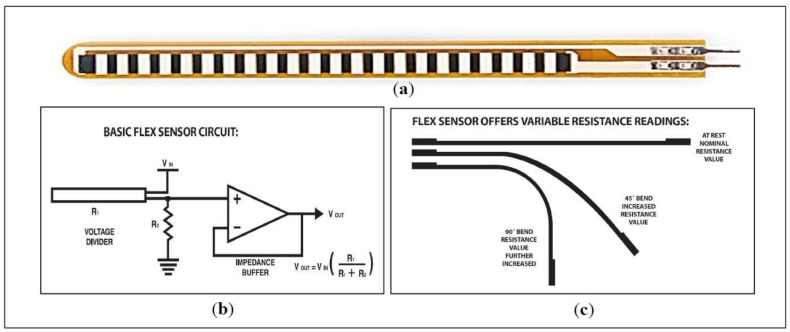
(**a**) Flex sensor, (**b**) flex bend levels, and (**c**)voltage divider circuit [2].

**Figure 7:**
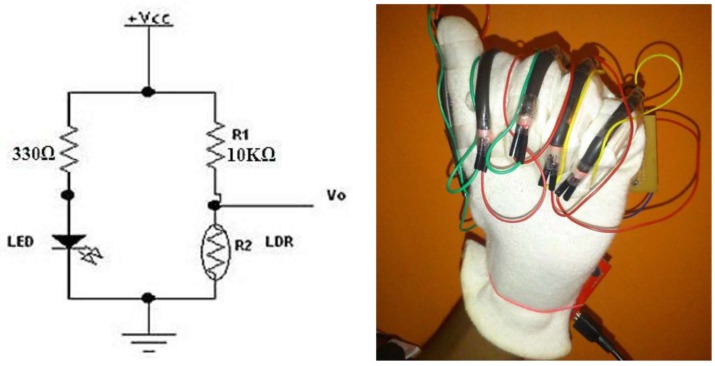
A circuit diagram of LED-LDR and the sensors’ positions on the glove [37].

The most prominent movement that can be performed by the four fingers (pinkie, ring, middle, and index) is bending towards the palm and then returning to the initial position. The thumb has unique advantages over the other fingers, thus enabling it to move freely in six degrees of freedom (DOF). In general, the predominant movement in SL related to fingers is bending. Finger tilt can be detected by using different methods, as shown in the literature. Flex sensor (Figure 6), which determines the amount of finger curvature used by a wide number of researchers and developers, is the most common of these kinds of sensors [21,37,38,39,40]. The Flex sensor technology is based on resistive carbon elements. When the substrate is bent, the sensor produces a resistance output correlated to the bend radius—the smaller the radius, the higher the resistance value. Thus, the resistance of the flex sensor increases as the body of the component bends. The flex sensor is very thin and light weight, so it is also very comfortable, and the most common available sizes are 2.2 inches and 4.5 inches. The price of the flex sensor is between $9 and $15, depending on the size. The optical sensor used to measure the angle of the finger curvature—in order to determine its shape by the amount of light passing through the channel—depends on the optic technology [41,42,43]. Optical sensors are electronic detectors that convert light, or a change in light, into an electronic signal. Furthermore, the combined pair of light-emitting diode-light dependent resistor (LED-LDR) is used (Figure 7), but not frequently, to detect bend of the finger. LDR is a component that has a (variable) resistance that changes with the light intensity that falls upon it [44]. Both of these optic technologies work on the measure of light intensity so that when the finger is straight, the density of received light will be very significant, and the opposite will be true when the finger bends. Both are suitable for handicapped individuals whose fingers can barely perform even very small motions.

**Figure 8:**
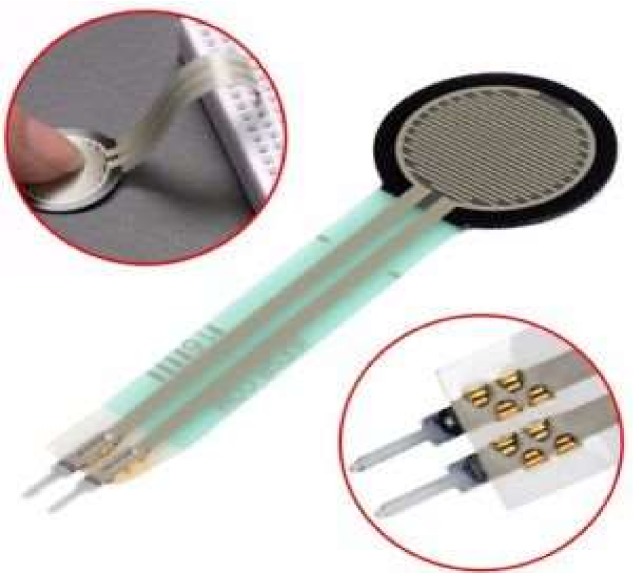
Tactile Sensor of 0.5 inch in size [8].

A tactile sensor (also known as a force sensitive resistor) is a robust polymer-thick film device whose resistance changes when a force is applied (Figure 8). This sensor can measure force between 1 kN to 100 kN. The tactile sensor resistance changes as more pressure or force is applied. When there is no pressure applied, the sensor looks like an open circuit, and as the pressure increases, the resistance decreases [35]. Thus, the tactile sensor is employed to calculate the amount of force placed on the finger, by which it can be determined whether the finger is either curved or straight [39,45]. A tactile sensor has a round, 0.16 inch, 0.5 inch, and 1 inch area diameter. The average cost of a tactile sensor is between $6 and $25.

**Figure 9:**
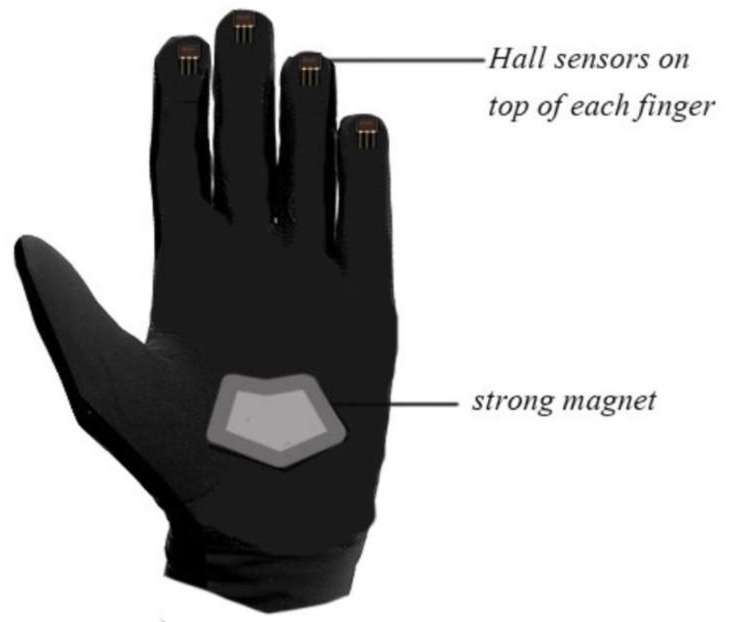
The sensory glove consists of Four Hall sensors on the tip of the four fingers [36].

Another principle was exploited for the purpose of figuring out the shape of the finger by an applied magnetic field and to determine the voltage variation over an electrical conductor. Accordingly, the above could be achieved through the use of Hall Effect Magnetic Sensor (HEMS). The unipolar Hall Effect sensor (MH183) used can detect the south poles of the magnet and is readily available. Effect sensors are placed on the tips of the fingers, and the magnet is placed on the palm with the south pole facing the top, as shown in Figure 9. When the South Pole is brought to the front face of the Hall sensor, it generates a 0.1–0.4V output. For the same purpose, other articles relied on the use of the ACC sensor technique to ascertain the shape of the fingers [4,11,14,46]. The sensor is lightweight with a high level of recognition accuracy, and has a low manufacturing cost.

b. Sensors Used to Detect Movement and the Orientation of the Hand

**Figure 10:**
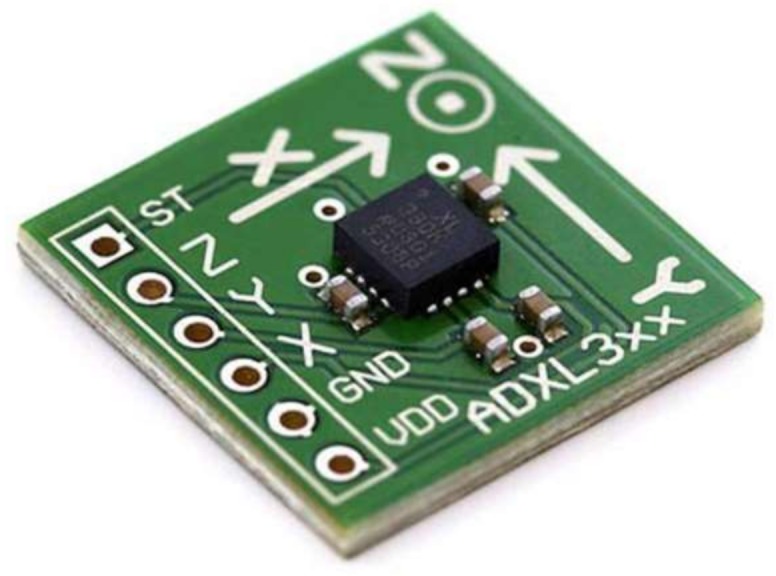
The ADXL335 3-axis ACC with a three-output analog pin x, y, and z [47].

Considering that SL postures are made up of hand and wrist movements, they should be considered. Despite the benefits of using sensors to determine the shape of the finger, hand movements cannot be distinguished by these sensors. The features of the ACC sensor enable it to distinguish the movement and rotation of the wrist as another factor in addition to its capability to determine the shape of the finger. Therefore, the three-axis ACC that supplies the differences in acceleration along every axis is used to capture the orientation and movement of the wrist to correctly represent the important function of a sensor glove. The ADXL335 (Adafruit Industries, New York, NY, USA) (Figure 10) is a thin, small, low power, complete 3-axis accelerometer with signal conditioned voltage outputs. It measures acceleration with a minimum full-scale range 3g. This device measures the static acceleration of gravity in tilt-sensing applications and dynamic acceleration resulting from motion, shock, or vibration. The ADXL335 contains a polysilicon surface micro machined structure built on top of a silicon wafer. Polysilicon springs suspend the structure over the surface of the wafer and provide resistance against acceleration forces. A differential capacitor, consisting of independent fixed plates attached to the moving mass, measures the deflection of the structure. Acceleration unbalances the capacitor, which, in turn, results in a sensor output with amplitude proportional to the acceleration experienced. The price varies from $9 to $24 depending on ACC version.

**Figure 11:**
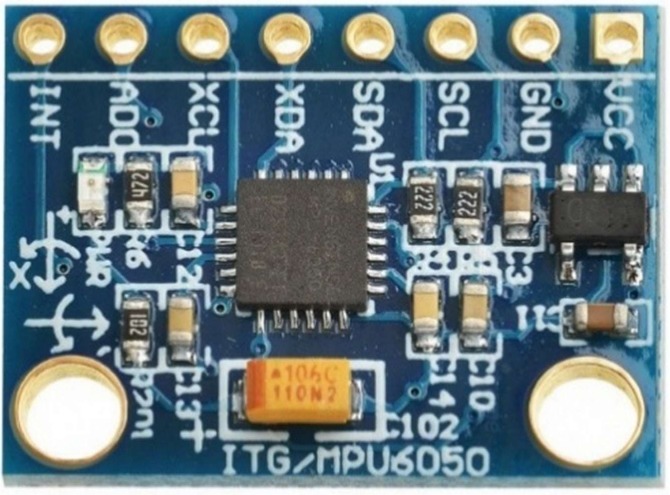
The six DoF IMU, MPU6050 chip consists of a 3-axis ACC and 3-axis gyroscope [50].

Combining a 3-axis ACC and a 3-axis gyroscope on the same board makes it suited for measuring tracked motion; the device presents data regarding accelerations in all three directions plus rotations around each axis [42,48,49]. Gyroscopes measure angular velocity and how fast something is spinning about an axis. If one is trying to monitor the orientation of an object in motion, an accelerometer may not provide enough information to know exactly how it is oriented. Unlike accelerometers, gyros are not affected by gravity, so they make a great complement to each other. One usually sees angular velocity represented in units of rotations per minute (RPM), or degrees per second (°/s). The three axes of rotation are either referenced as x, y, and z, or roll, pitch, and yaw. The IMU namely MPU6050 (InvenSense, San Jose, CA, USA) (Figure 11) is a 6 degree of freedom chip from Invensense company, containing a tri axis accelerometer and a tri axis gyroscope. It is operated off a 3.3V supply and communicates via I2C serial protocol at a maximum speed of 400 kHz. The accelerometer measurement is read by the microcontroller unit (MCU), using 16 -bitAnalog to Digital Conversion (ADC). The price of MPU6050 is about $30.

**Figure 12:**
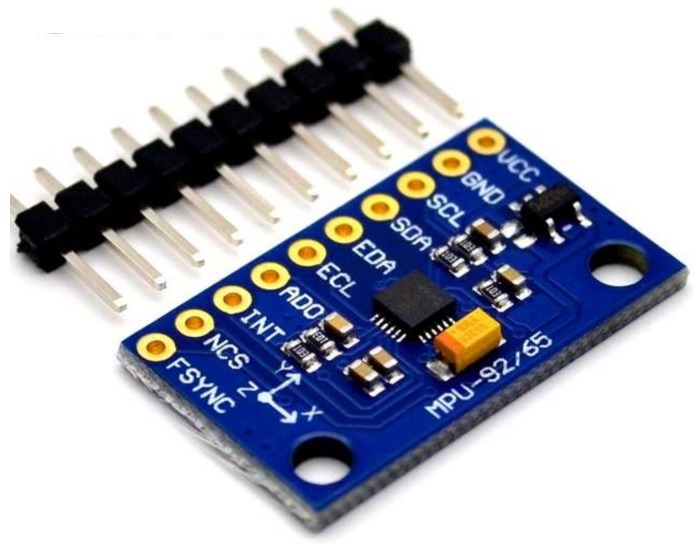
The 9 DoF IMU, MPU-9250 breakouts [53].

Furthermore, adding a 3-axis magnetometer to a 3-axis accelerometer and a 3-axis gyroscope will obtain a 9-axis inertial measurement unit (IMU) sensor which provides 9DoF plus roll, yaw, and pitch information during motion and orientation of the hand. Amagnetic field sensor is a small-scale microelectromechanical systems(MEMS) device for detecting and measuring magnetic fields (Magnetometer) [46,51,52]. The IMU version MPU-9250 (Figure 12) replaces the popular end of Life (EOL) MPU-9150 and decreases power consumption by 44 percent. According to InvenSense, “Gyro noise performance is 3× better, and compass full-scale range is over 4× better than competitive offerings.” The MPU-9250 uses 16-bit Analog-to-Digital Converters (ADCs) for digitizing all nine axes, making it a very stable 9 Degrees of Freedom board. The price of the MPU-9250 is about $15.

#### 2.1.2. Processing Unit

**Figure 13:**
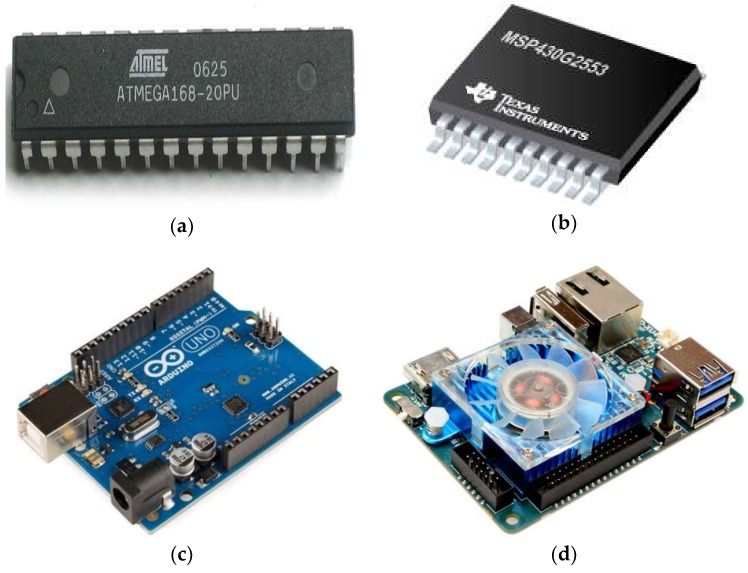
(**a**) ATmega microcontroller, (**b**) MSP430G2553 microcontroller, (**c**) Arduino Uno board, and (**d**) Odroid XU4 minicomputer.

The microcontroller is the system’s mind that is accountable for gathering the data from the sensors provided by the glove and performing the required processing of these data to recognize and transfer the sign to the output port to be presented in the final stage. A high-performance microcontroller with a microchip with 8-bit AVR microcontrollers based on reduced instruction set computer (RISC), which combines 32KB in-system programming (ISP) flash memory with read–write capabilities called ATmega (Figure 13a), was used in [8,9,35,50]. A micro-controller MSP430G2553 (TEXAS INSTRUMENTS, Dallas, TX, USA), (Figure 13b) with an 8-channel, 10-bit (analogue to digital converter) ADC was used [37]. The central processor modules ARM7 and ARM9 are used by [54,55], respectively. Furthermore, as found in the literature, an open-source electronics platform called Arduino has been used. Several Arduino boards are available on the market such as Arduino Nano, Uno, Mega, etc.; for instance, Arduino Uno (Arduino, Italy), (Figure 13c) is based on the ATmega328P microcontroller and has 14 digital inputs/outputs, 6 analog inputs, a 16 MHz quartz crystal, and USB connection [6,8,21,37,56,57,58,59]. Odroid XU4 (Hardkernel2, Anyang, South Korea) (Figure 13d), produced by Hardkernel2, uses a Samsung Exynos5 Octa 5410 processor that consists of 4 CPU cores each with Cortex™-A15 cores; it was used for the development of the data glove [59].

#### 2.1.3. Output Unit

Customarily, a user interacts with the device through output devices, so the output devices play an important role in achieving the best performance of devices implemented in the field of SLR. The main device adopted by researchers as the output was the screen of computer [3,4,35,40,48,60,61,62]. Other devices attracting the attention of researchers are the liquid-crystal display (LCD) [12,35,57,63], speaker [38,46,54,64], or both [6,51,55,65,66,67,68]. Ultimately, the smartphone is another alternative chosen for system output [9,42,48,59,69]. 

### 2.2. Gesture Learning Methods

Software, which is an essential component of every system, plays an important role in data processing in addition to the possibility of improving system outputs. The development of software for SLR systems is related to the methods used in the classification process to recognize gestures. One of the common direct methods to perform static posture recognition is prototype matching (also known as statistical template matching), which operates on the basis of statistics to determine closest match of acquired information values with pre-defined training samples called ‘templates’ [3]. In fact, this method is characterized by the lack of a need for complicated training processes or wide calibration, thus increasing its speed. From a pattern recognition standpoint, the artificial neural network (ANN) is the most popular method used for machine learning in the recognition field [67,70]. Therefore, it is possible to train this technique to distinguish both static and dynamic gestures, as well as posture classification, based on the data obtained from the data glove [41,43,54,70,71]. Long-term Fuzzy Logic has been used in many fields that need human decision-making; one of these areas is recognition of sign language [4,21,42,72]. Likewise, there is another useful algorithm; in fact, it falls within the scope of machine learning employed to provide accurate and less complicated classification through dimensionality reduction with improved clustering, which is known as Linear Discriminant Analysis (LDA) [46,61]. Hidden Markov Models (HMMs) is a popular technique that has shown its potential in numerous applications such as computer vision, speech recognition, molecular biology, and SLR [53,73,74,75,76]. Besides the HMM, the KNN is also used to classify hand gestures [63], and the KNN classifier with support vector machines (SVM) has been applied in posture classification in [77]. The KNN is applied in work on the recognition of ASL signs [40].

### 2.3. Training Datasets

**Figure 14:**
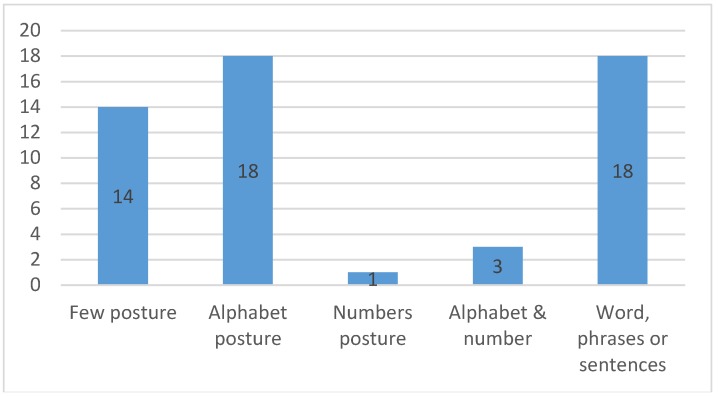
Number of articles on each variety of gestures.

The attention given to the sensory glove is due to its capability to capture the data required to photograph the shape and movement of the hand for the purpose of recognizing the gestures of SL. Despite the fact that SL has plenty of postures, many studies have concentrated on a selective set of postures that may be as small as some letters of the alphabet [21,44,47,51,78]; or the postures of the most frequently used words [37,41,45,55,56,72,77,79]; or a combination of the alphabet, numbers, and words [6] to perform their experiments and develop an SLR system. Others have contributed to the expansion of the database of gestures allocated for the purpose of developing a system to distinguish them and make them include either whole alphabets [4,11,35,46,50,57,64,65,70], numbers within the range 0 to 9 [10], or both, in the same system [39,42,69]. Furthermore, others contributed to their endeavor to distinguish certain words and phrases, even sentences, chosen to cover a wide range of real-life circumstances, such as family, shopping, education, sports, etc. [54,73,74,75,78,80,81,82,83]. Figure 14 illustrates the number of articles in each variety of the aforementioned gestures.

Table 1 shows further details regarding the database used in most of the previous studies, such as the type of gestures used with the frequency of conducting these gestures. In addition, the table clarifies who created the signs alongside the number of performers. The total numbers of samples used in the experiments are also given.

## 3. The Analysis Results

**Figure 15:**
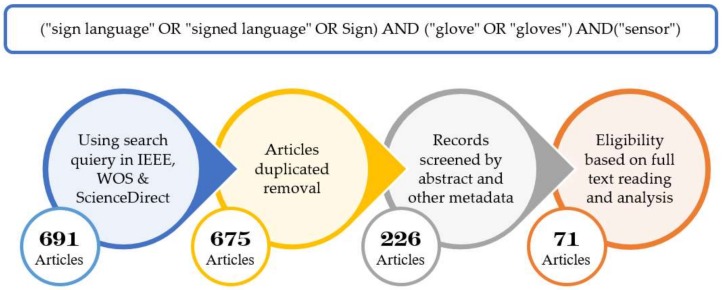
The searches query and article selection processes adopted in this study.

The databases, namely, Institute of Electrical and Electronics Engineers (IEEE), Web of Science (WOS), and ScienceDirect, have been adopted in this study, as they cover high-quality scientific publications (e.g., peer-reviewed journals, articles, conference proceedings, and workshop papers) related to computer science. Likewise, as suggested by [66], 691 articles, including 590 from ScienceDirect, 53 from IEEE Xplore, and 48 from Web of Science, were obtained as primary research findings. Sixteen duplicate records were eliminated through manual removal, thus yielding 675 unique papers for consideration. Screening of abstracts and metadata led to the further exclusion of 449 papers because they did not match any of our search queries. A total of 226 studies remained for full-text analysis. The analysis excluded 155 other records because of the failure to meet the inclusion criteria. Studies should be relevant to the development of an SLR system using a sensory glove, and should have accessible, online, full-text versions. Moreover, only publications written entirely in English have been accepted. Figure 15 shows the article selection process adopted in this study.

**Figure 16:**
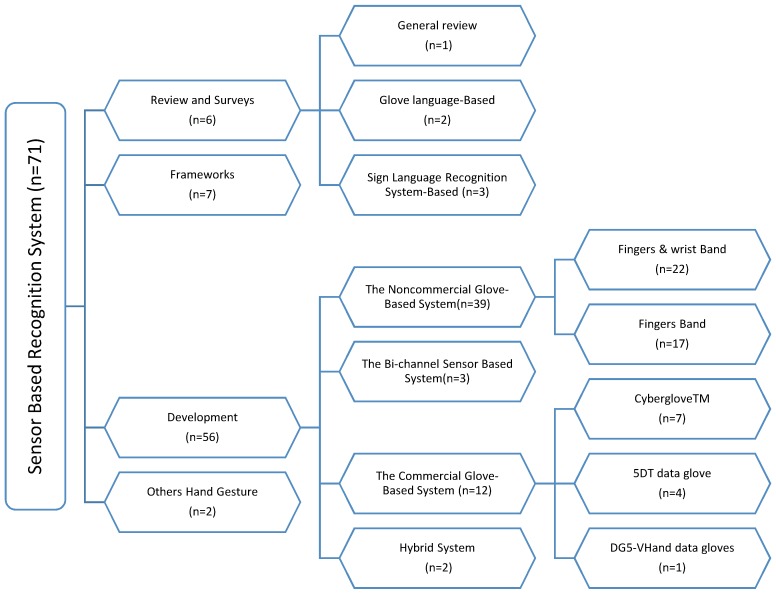
A taxonomy of literature concerning sensor-based sign language recognition.

In the final screening, 71 studies that satisfy the inclusion criteria were identified. The papers included in the identification of important items for each study were read numerous times, and all of this information was listed in a single Excel file. This information concerned the type of article, motivation, problems, obstacles, and challenges. Also identified was the deaf language targeted by the study with a focus on the types of sensors and tools used, as well as the methods and algorithms that were adopted in these studies. Most of the records (78.87%; 56/71) are development papers that refer to genuine endeavors to develop a recognition system or share expertise in doing so. Other papers (9.85%; 7/71) are proposals for models or frameworks to resolve the predicament of deaf and dumb people. A few articles (8.45%; 6/71) demonstrate existing work on recognizing a specific SL or general recognition system to investigate the desired features and disadvantages and the applications of new assistance tools. The smallest portion of studies (2.81%; 2/71) includes articles that focus on hand gesture detection and its applications in SLR. These patterns were observed to create four general categories of articles (review and survey, development, framework, and other hand gestures) and answer the questions that were initially posted. Thereafter, the groups were refined into the literature taxonomy (Figure 16). The main category was also divided into subcategories while ensuring that no overlap occurred. The observed categories are listed in the following sections.

### 3.1. Review and Survey Articles

The importance of review articles is well recognized among those who work in research that aims to absorb new phenomena, introduce them to the research community, and extract meta-statistics to understand the possibilities and effects of phenomena on instituting change. Despite the importance of these type of articles, among the 71 studies, only six belonged to the review and survey category, which consisted of three groups, namely, general review [84], SLR system-based [17,84,85], and glove-based [11,21]. General review papers provide a complete picture of the glove system used with an explanation of its applications. Moreover, this type of paper analyzes device characteristics, provides a roadmap for technology development, and addresses the limitations of existing technologies and trends in research boundaries. Articles related to the glove-based system review the development issues of several current systems that are used as an interpreter ofspecific SL to communicate with the deaf and dumb. Through these papers, a definite comparative approach is achieved for the selection of suitable technology and for the translator systems of a particular SL. A recognition-based system provides an overview of the research onSLR, analyzes the essential components of SL, captures several methods and the recognition techniques that may help to develop a sign language recognition system with a wide vocabulary, and highlights the strengths and weaknesses of the developed systems.

### 3.2. Development System for SL

The majority of studies (56/71 papers) obtained through the research process belonged to the development category. These articles were classified into different topics and applications. The selected studies were categorized into subcategories based on the types of gloves and sensors used.

#### 3.2.1. Non-Commercial Glove-Based System

Broadly defined, a non-commercial glove sensor is an electronic component, unit, or subsystem that distinguishes hand movements or changes in finger bending and sends data to other electronic devices, often to a computer processor. This sensor is developed and manufactured by self-effort. Most of the papers belonged to this category (39/71). The articles in this category can be distributed into the following two subcategories depending on the portion of the upper limb of the body that needs to be distinguished.

**Figure 17:**
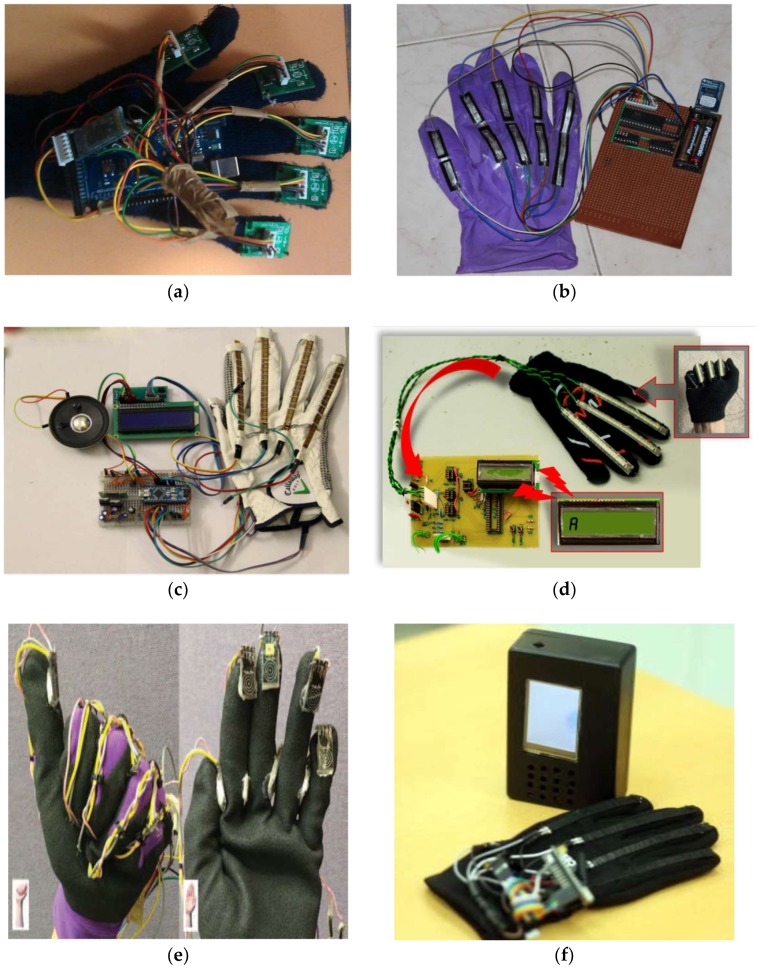
Finger bend detection glove used in the literature. (**a**) The glove consists of five 3-axis ACCs and (**b**) the ten custom-made flex sensors; (**c**) the translator system consists of five flex sensors mounted on glove, LCD and speaker;(**d**) the glove consists of three flex sensors used to detect few gestures; (**e**) the glove is equipped with five contact (pressure) sensors; (**f**) the wireless translator system is embedded with the five flex sensors and LCD.

First subcategory, finger band (17/39): All articles that are listed in this category are relevant to the development of a model, system, or device for gesture recognition via finger-bending detection. These articles reported the potential types of sensors that can be employed to determine the amount of finger curvature by identifying advantages and drawbacks. They also elucidate the dataset size that can be distinguished in addition to mentioning the accuracy achieved whilst implementing the system. Figure 17 lists samples of the developed glove, which can capture finger bend. Ten flex sensors were used to acquire finger bending data. Two sensors were attached to each of two joints over each finger. The high-speed, low-power analog Multiplexer (MPC506A, TEXAS INSTRUMENTS, Dallas, TX, USA) was used to collect 10 analog input signals from flex sensors in order to process data using the MSP430G2231 microcontroller (TEXAS INSTRUMENTS, Dallas, TX, USA). The code in microcontroller-received data from the Multiplexer was then arranged in the form of a packet with a marker character, and this packet was sent using an EZ430-RF2560 Bluetooth transceiver chipset (TEXAS INSTRUMENTS, Dallas, TX, USA) to a cellphone. The Bluetooth module is transmitting at a 11520 baud rate. Once the angles have been found using software installed on mobile, they are compared to the known poses from the database. Then, an error parameter is computed for each of these poses. Finally, a text to speech synthesizer is used to convert the gathered information to voice [44]. In [9], a gesture recognition glove was developed with 5 three-axis ADXL 335 ACC sensors. The ATmega 2560 microcontroller (Microchip Technology, Chandler, AZ, USA) decodes American Sign Language (ASL) gestures by considering the axis orientation with respect to gravity and their corresponding voltage. Via a Bluetooth module (SPP-TTL INTERFACE), the alphabet/word is sent to an android application to convert them into text and voice. A finger recognition system was presented to help handicapped persons express their intents by using a finger only. Five optical fiber sensors surrounded each finger. In order to represent the strength of the optical signals, 8 digits were used to measure each finger bend. The system used a 3-layered neural network module to learn the input gestures with the MATLAB program. The input nodes represent the chosen feature values; the hidden nodes use TANSIG (Tan-Sigmoid) as the transfer function. The Purelin (Linear) transfer function was used for one output node. The six different values served as outputs for the six predefined hand gestures. The Back-Propagation algorithm is used for training, and the tenfold validation method is applied to 792 records [7]. In [2], five flex sensors were mounted on the glove to recognize British and Indian sign language. The size of the flex sensors used was about 112 mm and about 0.43mm in thickness. The MSP430F149 microcontroller (TEXAS INSTRUMENTS, Dallas, TX, USA) compared gestures to equivalent electrical signals corresponding to memory storage. The valid word was displayed on the LCD and spoken out of the speaker. The sign to Letter Translator (S2L) system was developed as in [12]. The system consists of a glove, six flex sensors, discrete components, a microcontroller, and an LCD. Five flex sensors were mounted on each finger and one on the wrist. The analog inputs of six sensors are converted to two-bit zeros, one corresponding to each letter for the five flex sensors. The final output was produced via some ‘if’ conditions. An LED-LDR pair was used in [37] to detect finger bending (one pair for each finger). The MSP430G2553 microcontroller (TEXAS INSTRUMENTS, Dallas, TX, USA) converts the analog voltage values to digital samples and the ASCII code of the 10 English alphabet. The ZigBee Bluetooth was used to transmit recognized gestures. The received ASCII code is displayed on the computer, and the corresponding audio is played. The ELECTRONIC SPEAKING GLOVE was presented in [38]. The flex sensor was very thin and light weight (4.5 inches in size). The high-performance, low-power AVR^®^ 8-bit TMEGA32L Micro-controller with 10-bit ADC, RISC Architecture was used to recognize ASL alphabets via the template matching algorithm. The SpeakJet IC introduced the preconfigured 72 speech elements (allophones). In [41], the reliability of resistance sensors on the ASL translation via virtual image/interaction was investigated. Two flex sensors were used to carry out the experiment. The movement signal of the index finger and middle finger was sent to the Arduino Uno microcontroller. The system tested the six letters “A, B, C, D, F, and K”, as well as the number “8” using Fuzzy Logic via MATLAB software. The data of four gestures were acquired using a data glove equipped with five flex sensors. The change in resistance of flex sensors was fed into the Arduino Nano to implement the template matching algorithm in order to recognize the specific gesture and prerecorded audio command that was stored in the Secure Digital (SD) memory card; this was then transmitted through a speaker and display the text through a 16 × 2 LCD display [68]. The hardware structure of [63] consists of three flex sensors, an INA 126 instrumentation amplifier, an analog-to-digital converter (ADC), a 16F877A microcontroller, and an LCD. The sensors were placed on three fingers, namely, the ring, middle, and index fingers, to identify the letters of the alphabet from the finger spelling of ASL. Twenty-six characters of ASL were stored in the Programmable Intelligent Computer (PIC) microcontroller library. The obtained data of gestures were compared with 26 characters of ASL that were stored in the PIC’s micro-controller library, and the right letter was displayed on the LCD. The data glove successfully recognized up to 70% of ASL characters. The system proposed in [57] contained a pair of sensory gloves embedded with flex and contact sensors. Each glove consists of nine flex sensor, eight contact sensors, ATmega328P microcontrollers (Microchip Technology, Chandler, AZ, USA) and an XBee Bluetooth module (Digi International, Minnetonka, MN, USA). The flex sensors F1 to F5, namely, ‘outer flex sensors’, were used to calculate the bending changes on top of the five fingers, and the flex sensors F6 to F9, namely, ‘inner flex sensors’, were employed to detect the changes in orientation beneath the finger (excluding the thumb). A data set of 36 unique ASL gestures was used to evaluate the system; the recognition engine based on template matching resides in the master microcontroller, which processes the input and identifies the gesture. The system accuracy was from 83.1% to 94.5% with a cost of about $30. The ASL glove-based gesture recognition system is presented in [60]. The variation in electrical resistance of five custom-designed flex sensors was processed as the input to an Arduino ATMega328 microcontroller; then, it was compared with stored values corresponding to gestures. The system performance achieves accuracy of up to 80%. The cost of the system is approximately less than USD 5 in laboratory conditions using off-the-shelf components.

**Figure 18:**
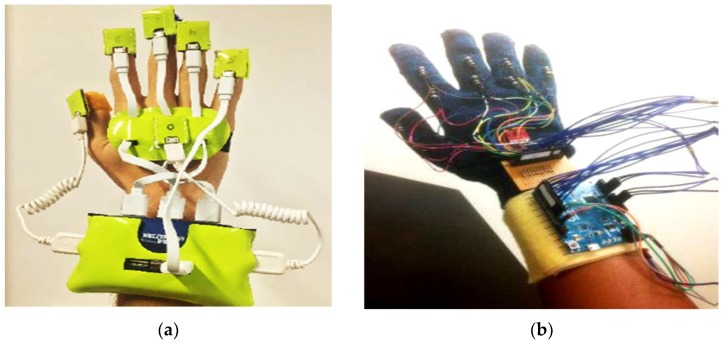

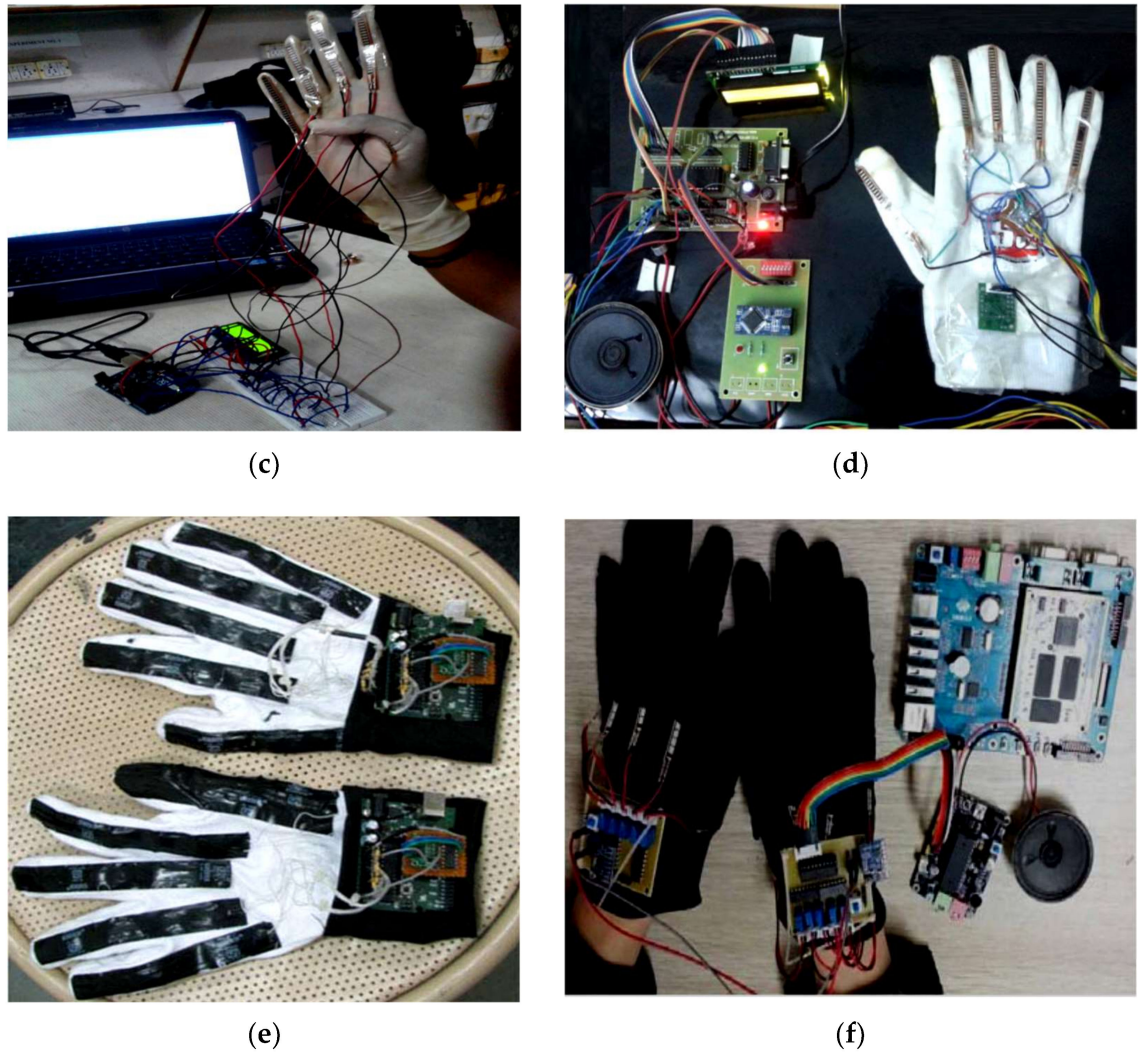
The prototype sensory glove for measuring finger bend and hand movement used in literature. (**a**) Six 9-DoF IMUs mounted on the glove for ArSL recognition; (**b**) a pair of optical sensors located on each finger and one ACC on the palm; (**c**) the translator system consists of five flex sensors placed on the back of each finger and ACC; (**d**) the data glove is embedded with five flex sensors, ACC, LCD, and speaker; (**e**) the system consists of two gloves equipped with ten custom-made flexion sensors and two ACC sensors; (**f**) the system consists of two gloves equipped with ten custom-made flexion sensors, two ACC sensors, and a speaker.

Second subcategory, fingers and wrist band (22/39): This category encompasses every study conducted to develop a model, system, or device for gesture recognition by detecting finger tilts and wrist orientation. These articles reviewed several types of sensors for detecting the movements of fingers and wrists. The advantages and disadvantages of recognition systems were also presented. The datasets and samples that were used were described, and the accuracy achieved whilst the system was implemented was stated. Figure 18 lists samples of the developed glove that can capture the flexion and abduction of fingers, as well as measuring hand motion. GesTALK, a standalone system, was presented to convert static gestures into speech [66]. The system can vocalize letters based on static gestures and can utter a string of words by concatenating words. The system vocalizes ASL and Pakistan Sign Language (PSL) letters expressed through static gestures made by the deaf individual. The glove consisted of 11 resistive elements, one for each finger and one for the abduction. Furthermore, the pitch and roll of the wrist were measured using two sensors. The system could recognize 24 out of 26 letters with an accuracy of 90%. A wireless sensory glove was developed in [4], as a new design of American SL to recognize the fingerspelling gesture. In addition to a 3D ACC, the glove had five 4.5-inch flex sensors and five contact sensors. The training data were collected from six speech-impaired subjects through ASL fingerspelling gestures. The system extracted 21 features from the sensors, and the experiment showed that the data glove can be enhanced by 12.3% using a simple multivariate Gaussian model. The glove-based system was proposed to track hand gestures. The system detects hand motions by using attitude and inertial measurements [59]. The prototype system consisted of six inertial measurement units (IMUs); five were at the upper-part of each finger, and one was on the wrist. Each IMU module provides 9-D ACC, gyroscope, and magnetometer measurements at a rate of 100 Hz, as well as attitude (orientation) estimated using an Extended Kalman Filter. The recognition process was carried out through Appling Linear Discriminant Analysis (LDA) on input signals sent to an embedded Odroid XU4 computer using a USB hub. The system achieved 85% accuracy. AcceleGlove, which comprises six ADXL202, ACC sensors was developed for Vietnamese SL posture recognition [58]. The system was modeled for the 23 Vietnamese alphabets, as well as two postures for “space” and “punctuation”. The data collected from the IMUs related to wrist and fingers joints. The fuzzy rule-based system was used to perform the gesture classification process. Twenty out of the 23 letters achieved 100% recognition, and the average system accuracy reached 92%. An intelligent glove was developed to capture the postures of the hand and translate these postures into simple text depending on the data acquisition and control system [50]. Three types of sensors were used in this data glove prototype: five flex sensors, five force sensors, one mounted on a finger, six DoF MPU6050 chip, tri axis ACC, and tri axis gyroscope sensors. All of these sensors were connected to Arduino mega, and the result was displayed on an LCD or smart phone. The electronic system could recognize the ASL alphabet (20 out of 26 letters) and achieve an average accuracy of 96%. A holistic approach was also presented to develop a real-time smart glove system to convert static SL gestures into speech by using statistical template matching [3]. The “Sign Language Trainer & Voice Convertor” software acquired five custom-made flex sensors and one 3-axis ACC along with an Arduino Duemilanove Microcontroller Board.

A haptic glove was presented as a vital means to acquire data from different hand signs, and the LabVIEW program served as the user interface with the aid of Arduino platform [47]. Arduino Mega was employed to collect gesture signals from the five custom-made flex sensors, contact sensors, and one ADXL335 ACC, placed on the glove. The flex sensors were mounted on each finger, and the contact sensors were placed between each adjacent finger. An electronic glove was equipped with five flex sensors, an ACC, and a contact sensor to recognize static and dynamic gestures and convert these gestures into visual information on an LCD, voice through a speaker, or both [67]. The embedded system is composed of data gloves with flex sensors and an ACC to detect finger tilt and hand rotation; thereafter, these signs are processed by a microcontroller, and the playback voice is the output of the system [79]. The glove maps the hand orientation and bending of fingers with the help of an ACC and Hall Effect sensors. The device is modelled to transform SL gestures to textual messages [36]. Four Hall Effect sensors (MH183), which were placed on all fingers except the thumbs, were used to detect the south poles of the magnet placed on the palm in order to measure finger tilt. The ADXL535 was also included to record the hand orientation via the triplet of x, y, z axes (voltages) information. The data acquired from the glove was passed to MATLAB script to detect the number of gestures (0–9) performed by the user and the accuracy of the recognition (about 96%). In another study, two elements, namely, the 3D positioning method and optical detection technique, were used to create a low-cost glove to interpret gestures [56]. A set of Light-emitting diode (LED) had an emission peak of 940 nm (model LTE-4602), a compatible photodiode (same model), and a polymeric fiber (PMMA). These optical sensors were used to capture finger tilt and the LSM330DLC chip; 3-axis ACC and 3-axis gyroscope were used to determine hand motion. A translator system was presented to interpret Urdu SL gestures by using a wearable data glove [6]. Five flex sensors of 4.5-inches in length were placed on each finger, and an accelerometer MMA 7361 (SparkFun Electronics, Niwot, CO, USA), was placed on the palm. The glove also contained Arduino Mega 2560, drive Graphical Liquid Crystal Display (GLCD), and playback module WTV020 SD. The Principal Component Analysis (PCA) was selected to obtain coefficients of the training set and create a 300 × 8 (m × n) data matrix. These matrices of coefficients were used to transform both the saved mean of sensor values for each gesture and real-time input components. The PCA was performed using MATLAB 7. The system performance reached 90% accuracy. The glove-based approach is used for hand movement recognition systems. Thus, the approach can recognize several words, numbers, and the alphabet of the Malaysian SL when the speaker wears the glove with 10 tilt sensors (two sensors for each finger); a 3-axis ACC to determine the motion of the hand via roll, pitch, and yaw values; and a microcontroller and Bluetooth module to send the converted data to a cellphone. The system was tested on a few gestures of Malaysian Sign Language using a matching template, and the accuracies ranged from 78.33% to 95% [69]. Twenty-six hand gestures correspond to the letters of the alphabets, and 10 other gestures represent numbers, which can be recognized by the data glove model presented in this work [51]. Five flex sensors were used, one on each finger and thumb fed to the Arduino uno via an analog pin (A0 to A4). In order to determine the palm tilt, a ADXL335 ACC was used, and 2^8^ (256) gestures were the system’s capacity to store learned gestures for the matching process. Combining flex sensors with nine degrees of freedom, ACC sensor and ARM9 allowed the degree of finger bending and hand orientation to be measured [54]. Therefore, this combination can distinguish several SL postures with the assistance of the matching method. The system consists of two gloves; each glove contained five 4.5 flex sensors of 95 millimeters in length. Additionally, one 9-DoF IMU was used to detect the orientation and movement of the palm. The system was tested with the phonetic alphabet such as a, b, c, zh, ch, and other phonetic letters with an average accuracy of more than 88%.

#### 3.2.2. Commercial Glove-Based System

**Figure 19:**
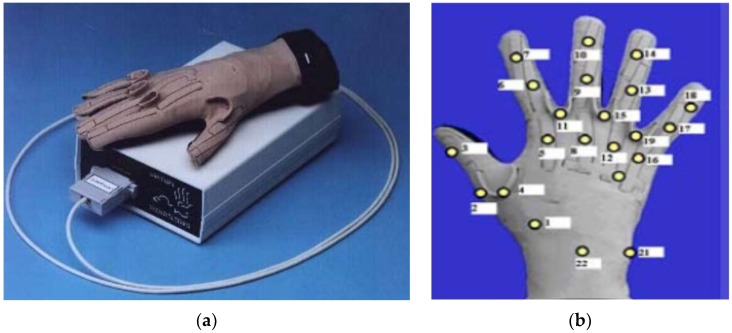
Glove systems. (**a**) Virtual Technologies’ CyberGlove and control box and (**b**) the location of 22 bend sensors on the glove [43].

**Figure 20:**
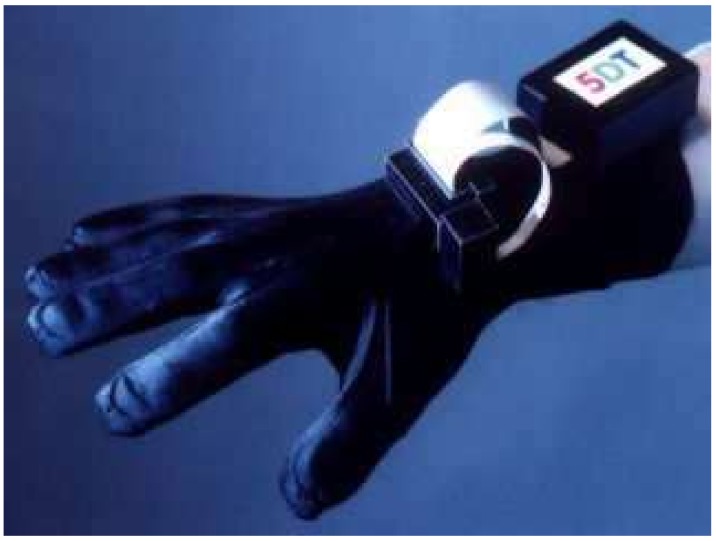
The 5DT Data GloveTM developed by Fifth Dimension Technologies; the glove measures seven DOF [74].

The commercial glove-based system is a means to handle the quandary of communication for deaf and mute individuals. The system uses bend-sensing technology to capture hand and finger actions into digital joint-angle measurements. Several studies (12/71 papers) used different commercial gloves to recognize different SLs. Some of them (7/12) used a popular commercial glove called CyberGlove (GLV) (Figure 19). The lightweight elastic glove is equipped with 22 thin and flexible sensors that are virtually undetectable. The glove consists of three sensors attached to each finger, four abduction sensors between fingers, a palm-arch sensor, and sensors to measure wrist flexion and abduction. The price is approximately $40,000 per pair for 22-sensor Cybergloves. An ASL translator (into written English words) was developed using artificial neural networks (ANN). The systems, based on the CybergloveTM glove and a Flock of Birds 3-D motion tracker, were used to capture the flexion of fingers and trajectory of the hand. The sensors data were processed by two neural networks, namely, a word recognition network and velocity network, with 60 ASL words. The recognition accuracy of these two systems is 92% and 95%, respectively [81]. A 3D hand model was designed to display posture related to reconditioned words. A 3D hand model was constructed using java 3D. The system segmented and recognized the continuous words, which were recorded using CyberGlove in real-time. The recognition efficiency was tested using the index tree [43]. A CybergloveTM and a FlockofBirdss 3-D motion tracker are used to collect hand motion and bending. The 15 bending angles, three positions, and three orientation readings from the Flock of Birds were classified using a multi-layer neural network. The ANN consists of 151 input variables, 100 hidden neurons, and 50 output neurons. A Levenberg–Marquardt back propagation algorithm was used for training 50 words in ASL, and the system reached about 90% recognition accuracy [74]. A segment-based probabilistic was presented to detect and recognize the continuous gestures of ASL. A Bayesian network (BN) was used to segment the continuous signs. A two-layer conditional random field (CRF), along with support vector machine (SVM) classifiers, were applied for the purpose of recognition. The system performance had average accuracy of 89% [61]. A new approach was proposed by fusing the data acquired from sensor gloves, and the hand-tracking system used the Dempster–Shafer theory for evidence. The recognition performance of 100 two-handed ArSL achieved an accuracy of about 96.2% [86]. The CyberGlove was used to collected finger tilt and hand motion. A linear decision tree with Fisher’s linear discriminant (FLD) was used to classify 27 Signing Exact English (SEE) handshapes. Hand movement trajectory was classified using vector quantization principal component analysis (VQPCA). The system yielded recognition accuracy up to 96.1% [87]. Moreover, a few (4/12) experimented with another commercial data glove known as the 5DT data glove to develop their SLR systems. The 5DT Data Glove Ultra is available in two variants, with five or 14 sensors per glove. The 5DT Data Glove 5 Ultra (Figure 20) is equipped with five fiber optic sensors to measure the flexion and one extra to measure the orientation (pitch and roll) hand. The 5DT Data Glove 14 Ultra consists of 14 fiber optic sensors (two sensors per finger), as well as the abduction between fingers. The glove is also equipped with two tilt sensors to measure the pitch and roll of the hand. The cost of one glove is approximately $995. A 3D model for finger and hand motion detection was developed to motivate the creation of a learning system based on gloves. The system used 5DT data glove 14 ultra-motion to capture Japanese fingerspelling gestures. This system helps a learner to recognize motion errors intuitively [70]. The manuscript presented software development to assist individuals suffering from speech and hearing impairment depending on the tuning location of the fingers with 5DT gloves. The 5DT Glove with five sensors was used to translate hand signs into words and phrases. Gesture recognition was performed using a multi-layered neural network. The NN configuration consists of one input layer (five input neurons), three hidden layers, and one output layer (twenty-six output neurons). Different algorithms, namely, back propagation, resilient propagation, quick propagation, scaled conjugated gradient, and Manhattan propagation, were used to train the network using the Matlab program [82]. The real-time architecture was presented to recognize Spanish SL in terms of gesture and motion recognition. A 5DT Data Glove 14 Ultra was used for real-time sign recognition. A distance-based hierarchical classifier has been proposed to recognize 30 signs of the Spanish alphabet [78].

**Figure 21:**
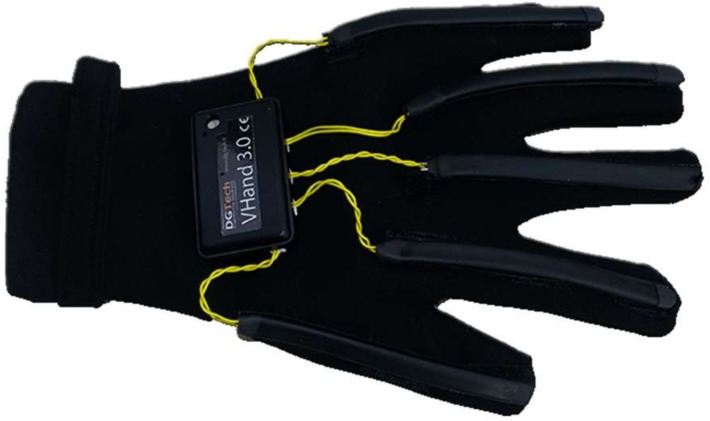
The A DG5-VHand glove equipped with five flex sensors and one 3-axis ACC [49].

Only one work (1/12) in this literature review adopted the DG5 VHand data glove in the task implementation. Two real-time translators were developed for American SL. A DG5-VHand glove (Figure 21) was equipped with five bend sensors and a 3-axis ACC, which allows for sensing both the hand movements and the hand orientation. The gloves are suitable for wireless operations and are powered with a battery. The device costs about $750 for a wireless left-hand glove. A novel technique classified sequential data for Arabic SL using a pair of DG5 VHand gloves. The Modified k-Nearest Neighbor (MKNN) was used for the classification of a sensor-based dataset. The dataset consisted of 40 sentences, which were captured using two DG5-VHand gloves. The performance of the proposed solution reached 98.9% recognition accuracy [40].

#### 3.2.3. Bi-Channel Sensor-Based System

Transacting with multimodal sensors by fusing data from various channels is not required to fully understand the connotations of sign posture. Therefore, hand posture recognition based on data fusion of multi-channel electromyography (EMG) and inertial sensors, such as a three-axis ACC, was proposed. The possible benefits of the combination of ACC and EMG signals are utilized to achieve multiple degrees of hand freedom [77]. A decision tree and multi stream hidden Markov were used to classify 72 Chinese Sign Language (CSL) words. The MMA7361, 3-axis ACC was mounted on the back of the forearm near the wrist to capture information about hand orientations and trajectories. The EMG sensors were located over five sites on the surface of the forearm muscles. The overall recognition rate of the system was up to 72.5%. The sensing system for German SL with a small database was investigated by using a single channel of single electromagnetism (EMG) and single acceleration (ACC) to recognize signs for seven-word level vocabularies [83]. The experiment was conducted on seven sign gestures (70 samples from each subject), with the k-Nearest Neighbor (k-NN, with k = 5) classifier and SVM. The system achieved an average accuracy of 99.82% for subject-dependent recognition and 88.75% for the general condition. Information from the three-axis ACC and five-channel electromyogram of the signer’s hand was analyzed using intrinsic-mode entropy towards automatic Greek SLR. An experiment was conducted on a 60-word lexicon with three native signers and repeated 10 times. The system used intrinsic-mode entropy (IMEn) by using a PC along with MATLAB R2007a. The experiment showed that the recognition rate reached 93% classification accuracy [76].

#### 3.2.4. Hybrid System for SLR

A small category includes papers associated with combining vision- and glove-based approaches for acquiring SLR data. The hybrid system was proposed to take advantage of the use of inertial sensor measurements and contribute to enhancing the value of acquired vision data. The system based on RGB color model (Red, Green and Blue) cameras or depth sensors and the Accelerometer Glove is presented in [53]. This issue needs further investigation. The Accelerometer Glove contained seven IMU sensors, five located on the fingers (one sensor on each finger), one on the wrist, and one on the arm. The database was created with 10 repetitions of each of the 40 gestures registered for the five signers. This results in a total of 2000 recordings used to validate the solution. The recognition engine was based on sign language gestures by using Parallel Hidden Markov Models (PaHMM). The system accuracy reached 99.75%. The prototype for a one-way SL translator consisted of AcceleGlove and a camera mounted on a hat. Acceleglove was equipped with five 2-axis ACCs located on rings to capture finger flexion. Two more on the back of the palm read the hand orientation. Another two ACCs were used to detect the bend angles of the shoulder and elbow along with the upper arm. The users perform 665 sentences of ASL, with a camera filming the process to aid in the identification of incorrect signs. In order to train the system, a HMM-based recognizer carried out phrase-level sign recognition with a per sign accuracy of 94% [52].

### 3.3. Frameworks for SLR

Several of the obtained articles (7/71) fit the developed category concerning our taxonomy, because they did not develop a new system. Rather, they (3/7) served as an eligible framework of the template [48,49,52]. Other articles (3/7) addressed the system design [42,73,75], whereas one article presented the development and use of a method or technique for the SL system [72]. In [42], a framework was proposed for wireless glove-based two hands gesture recognition. The framework was established for the real-time translation of Taiwanese sign language. Flex and inertial sensors were embedded into each glove. The bending of fingers was acquired by flex sensors, the palm orientation was acquired by G-sensor, and the gyroscope was used to obtain the motion trajectory of the hand. The sampled signal will be sent to a cell phone via Bluetooth. In the cellphone, the encoded digital signal is compared with the SL database using a lookup table, and the meaning of a valid gesture is displayed. Using Google translator, the cell phone produces a voice. Through the proposed architecture and algorithm, the recognition accuracy will be acceptable. In [49], the framework for a sensory glove for Arabic sign language signs (ArSL) is presented. The glove design is based on statistical analysis for all words of ArSL in terms of a single hand via as few sensors as possible. The proposed design was implemented using the PROTEUS simulation program. The system consists of two gloves; each glove contains six flex sensors (length of sensor = 5 cm), one for each finger plus one for the palm, four contact sensors for fingers (index, ring, middle, and pinky) and one MPU 6050 for hand orientation and motion. In [48], the framework consists of two algorithms: sign descriptor stream segmentation and text auto-correction; it also presents a hand gesture descriptor to develop the software architecture of this time-sensitive complex application. The motion-capture gloves produce text and audible speech. A framework of the Interpreter Glove system was proposed in [48]. The framework consists of four main blocks, namely, the glove, the training environment, the mobile applications, and the backend server. The system produced a gesture descriptor, namely, “Hagdil”, based on the structure and kinematics of the human hand. A Hagdil descriptor stores all the substantial characteristics of the human hand: parallel and perpendicular positions of the fingers relative to the palm, wrist position, and absolute position of the hand. Then, it encodes the Hagdil gestures descriptor (18 hand parameters) before transmitting it to the cellphone. Finally, the Levenshtein distance calculation algorithm is employed for auto-correction for text that corresponds to the gesture.

### 3.4. Other Hand Gesture Recognition

**Figure 22:**
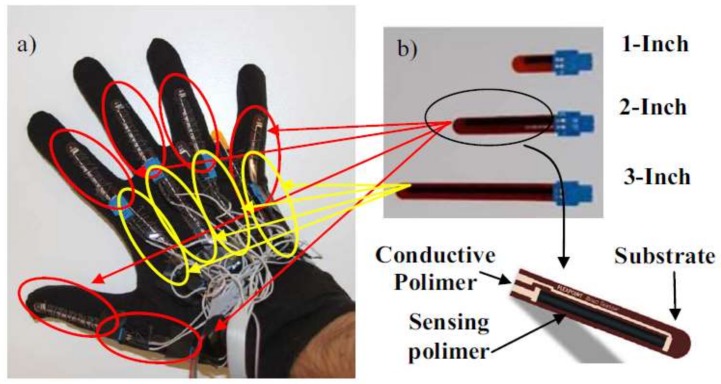
Adopted bent sensors and positions. (**a**) the image of the sensorized glove and (**b**) the adopted bent sensors [14].

The progress of science and technology has facilitated the development of hand movement detection devices. Gesture recognition is a system that translates hand movement through software, hardware, or a mixture of both to help hearing- and speech-impaired individuals communicate with a machine. The recognition system can support disabled people, medical staff, scientists, and others. One study [14] presented a simple, low-power, low-cost system that could detect finger flexion–extension movements. The glove consists of ten resistors (two for each finger) connected to a front-end microcontroller, which send the acquired data to the personal computer (PC)through the Bluetooth module (Figure 22).

**Figure 23:**
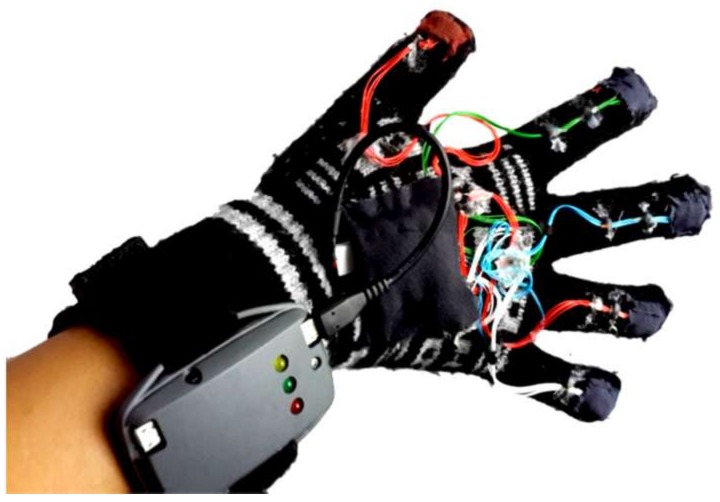
Six sensor-type 3-axis ACC mounted on a data glove for gesture recognition [88].

Five gestures, namely, rock, paper, scissor, rest, and thumb-up, were recognized using the glove equipped with six 3-axis ACCs. One ACC was placed on each finger to measure fingers bend, and one was placed on the back of the palm to capture hand motions and positions (Figure 23). The six ACCs were connected to a microcontroller, and the received raw data are mapped and arranged in an array before it is transferred serially to a Bluetooth module [88].

## 4. Distribution Results

Three digital databases were chosen to obtain information from works related to SLR based on a sensory glove. The outcomes of the review are classified in four general categories, namely, development, framework, review, and survey, and the last category is other hand gesture. The final number of articles gathered from IEEE Xplore is 35, composed of 24 articles for development, 4 for framework, 5 for review and survey, and 2 for other hand gesture. The closing number of articles gathered from the WoS database is 26, composed of 23 articles for development, 2 for framework, 1 for review and survey, and 0 for other hand gesture. Finally, the closing number of articles gathered from ScienceDirect is 10, composed of 9 articles for development, 1 for framework, 0 for review and survey, and 0 for other hand gesture. Table 2 lists the citations and Impact Factor of the cited papers in this study ordered by number of citations. The citation number of articles was found using Google Scholar, and the impact factor of the journals was extracted from the official website of each journal.

### 4.1. Distribution by Sign Language Nationality

**Figure 24:**
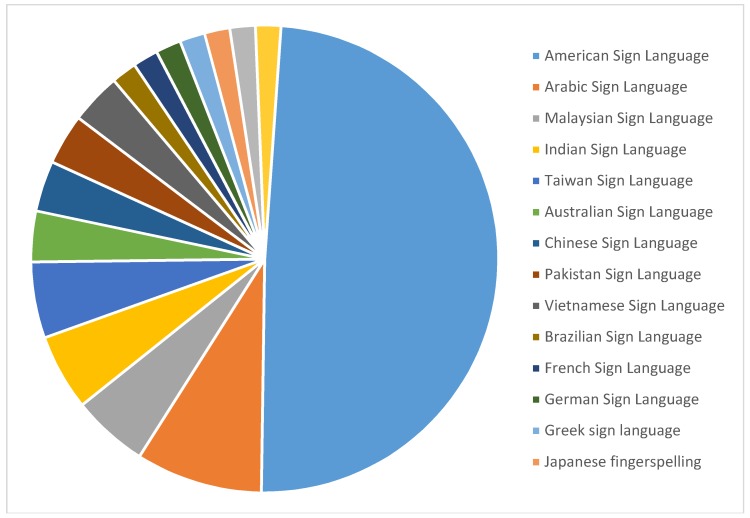
Number of articles published for each form of SL.

Figure 24 illustrates the number of articles on SL (16) that were included in this study. The most prominent SLs that have been mentioned are ASL (28 articles), ArSl (5 articles), MSL (3 articles), Indian sign language (ISL) (3 articles), and Taiwan sign language (TSL) (3 articles). However, in this study, only one or two articles were published for other SLs. ASL gained the most attention from researchers conducting their scientific studies in the field of gesture recognition. The number of research articles submitted in this area during the previous ten years is 28, which equates to 48%.

### 4.2. Distribution by Gesture Type

**Figure 25:**
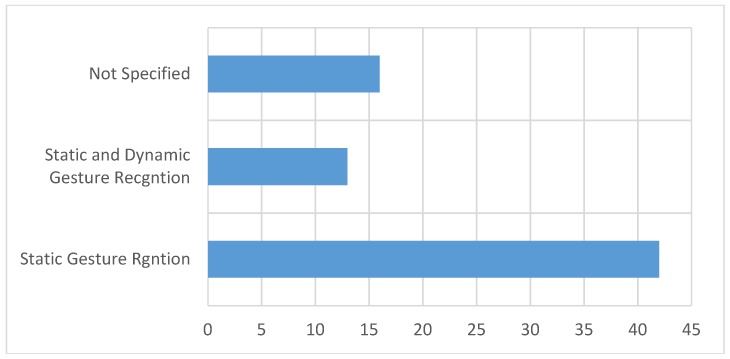
Number of articles that recognize static and dynamic gestures.

The SL similar to vocal language is composed of alphabets, words, and sentences, all of which are gesture expressions that may be static or dynamic. Somehow, the static gesture is less complicated than the dynamic one. Figure 25 shows that a large number of researchers (42/71) have developed a mechanism to distinguish static gestures, whereas few researchers (13/71) have developed a system to recognize dynamic gestures. Our review of the research work found that a number of other studies (16/71) did not distinguish the type of gesture targeted in their work.

### 4.3. Distribution by Number of Hands

**Figure 26:**
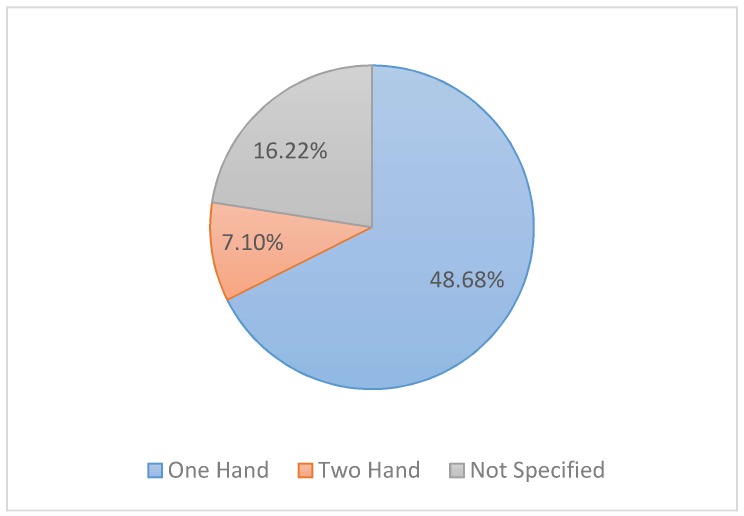
Number of articles for gesture recognition based on the number of hands.

Figure 26 illustrates the number of articles that executed gestures with one or both hands. It is easy to note the significant difference between the number of studies (48/71 papers) on the recognition of one hand and the number of studies (7/71 papers) on the recognition of two hands.

## 5. Discussion

This review provides a research-oriented overview of relevant studies on the latest achievements in the recognition of SL based on sensory gloves. The aim of this study is to clarify and highlight the potential research trends in this field. To achieve this goal, a taxonomy of relevant articles is proposed. Developing a taxonomy of the literature in the field of SLR offers many advantages. One of these benefits is to organize a variety of published work in a taxonomy to serve new SLR researchers who may feel confused with the large number of papers written on the subject without any kind of structure. Various studies discussed the topic from an introductory perspective, whereas other studies developed recognition techniques to obtain improved performance using existing commercial gloves. Several articles developed an actual data glove for SLR. The taxonomy of relevant articles sorts out various studies into a meaningful, manageable, and cohesive structure. Moreover, several important insights into the topic can be presented to researchers through the structure introduced by the taxonomy. One of these insights outlines possible research trends in this area. For example, the taxonomy in the current work on SLR based on sensory gloves shows that researchers tend to develop their own gloves and conduct experiments to offer a possible way of contributing to this area. Other areas include developing commercial gloves and techniques to procure the desired accuracy in the recognition process. A taxonomy may also reveal gaps in the literature. Organizing studies in literature into distinct classes highlights the weak and strong features of SLR in terms of research coverage. For example, the taxonomy in this study shows how groups of individuals worked to develop a glove using different types of sensors or investigated the recognition method using existing gloves and received notable attention in the development category (this phenomenon is evident as the bulk of the work falls in this category). Furthermore, the taxonomy shows the scarcity of studies on the development of a hybrid system to obtain data. The taxonomy also elucidates that studies in the development category focused on obtaining data on finger and wrist movements and did not attempt to include data on arm and shoulder movements, which are also related to SL signals. In addition, the subcategories of the taxonomy that belong to the survey and review branches have not received considerable attention in the literature. Three aspects are revealed in the literature review of this survey: the motivations behind SLR based on the sensory glove, the challenges in the appropriate use of these technologies, and the recommendations for alleviating these difficulties.

### 5.1. Motivations and Benefits of SLR Using Gloves

**Figure 27:**
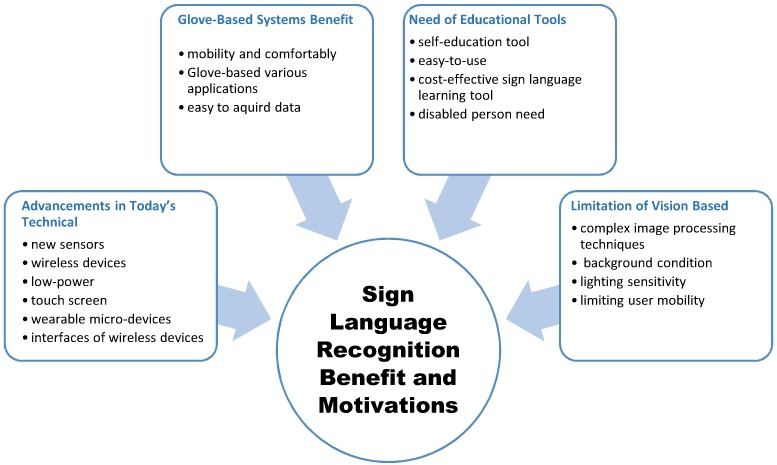
Categories of benefits of SLR based on the sensor approach.

When humans talk to one another, they communicate through speech and gestures. Gestures may either supplement one’s speech or completely change it, especially in the case of hearing- and speech-impaired individuals. However, these disabled people have overwhelming difficulty in communicating with others and find themselves in awkward situations, because various gestures may be used by several people to convey the same message.The following are some of the benefits and motivations reported in the references that have been categorized according to the similar motives (Figure 27).

#### 5.1.1. Advancements in Today’s Technology

The devices and tools that help disabled people live a normal and comfortable life have always been a domain that has attracted innovation. Various developments in the technological world today have helped diverse groups of people with disabilities in terms of research and product system development in assistive technologies that aid various disabled people to carry out their daily activities [3]. Such examples include wireless devices, low-power electronics, and the capability to design both the analogue front-end and digital processing back-end. Furthermore, integrated circuits have also inspired the production of a new range of micro wearable devices [44,58]. The interfaces of wireless devices available to us have become easier to use. Actually, most new wireless devices use a touchscreen instead of buttons [56]. This innovative interface can be more sophisticated with the integration of a system that is constantly aware of hand movements in space [56]. The interfaces of wireless devices available to us have become easy to use. This is also true in the development in the field of electronics in which many sensors can address bending measurement and recognize many degrees of freedom (DOF) of the human hand and its wide scope of movement. Similar new technologies can be used for various areas, such as SL translation [7,9]. Appropriate technology can be used to give a tremendous boost to the everyday life of deaf and dumb people and enhance the quality of their lives.

#### 5.1.2. Educational Tools for SL

In developing countries, deaf and dumb children rarely receive education. In addition, adults with hearing and speech loss have a significant unemployment rate. The availability of an easy-to-use and cost-effective SL learning tool will help these individuals learn through natural means. Therefore, improving access to education and vocational rehabilitation services will reduce unemployment rates for people with hearing or speech disabilities [81]. On the other hand, SL is the main tool to communicate with the deaf and dumb. Thus, many normal people are encouraged to begin to learn SL through web sites, video clips, and mobile applications. With respect to gesture (arms, hands, and fingers) skill learning, which is used in SL, a specialist should provide objective advice to the learner [9]. In other words, the learner requires a self-education tool to instruct her/him on how to do the correct gesture, follow up by implementing that movement, and identify errors that may occur during implementation [70]. The SL glove appears to be very beneficial in assisting sign language education.

#### 5.1.3. Advantages of Glove-Based Systems

Our hands are used to accomplish a large number of basic tasks in our daily lives. Thus, many studies have examined the development of techniques to investigate hand movements and increase the capability to simulate hand functions for the completion of basic tasks. Data acquired from hand movements are used in several engineering applications ranging from motion analysis to biomedical science [89]. Glove-based systems are important techniques that are used to obtain hand movement data [57]. Despite them having been around for more than three decades, this field of research is still extremely active [87]. It is also obvious that technological advancements in computing, materials, sensors, and processing-classification methods will contribute to making the new generation of glove systems more powerful, highly accurate, comfortable, cheap, and possible to use in many applications [36,66]. A glove can be an assistive interpreter tool for hearing- and speech-impaired persons to communicate with non-disabled individuals who do not understand SL [39]. Gloves have other benefits, such as *mobility and comfort*. Modern technologies and sophisticated electronic circuits, which are harnessed to recognize SL using gloves, have enabled gloves to overcome the need to be connected with a computer. Furthermore, these devices being lightweight makes it possible to carry them with ease and comfort [7,8]. Glove-based systems are also beneficial to various applications. New technologies for the man–machine interface (MMI) present natural ways to operate, control, and interact with machines. The MMI term refers to the capture and conversion of signals related to the appearance, behavior, or physiology of a human via a computer system [15]. All interface techniques that rely on sound or vision have contributed to a radical change in the mechanism of operating a computer [78]. Gesture recognition is an interaction method that plays an important role because of its common use of gestures and signs for human communication [4]. This fact increases the importance of recognizing a gesture, indicating that it is a growing field of research with incalculable application areas [82]. Numerous kinds of applications are currently involved in gesture recognition systems, such as SLR, substitutional computer interfaces, socially assistive robotics, immersive gaming, virtual objects, remote control, medicine-health care, etc. [10,11,12,13,14].

#### 5.1.4. Limitation of the Vision-Based Method

Gesture recognition systems rely on two means to capture and record the shape and movement of the hand: they use a camera or a sensor. In gesture recognition systems, the use of a vision approach requires the adoption of at least one camera to capture hand images and interpret the suitable gesture. Sometimes, several devices, such as Kinect, leap motion, or even a colored glove, are appended to increase the capability to detect hand shape or movements [85,90]. The advantage of this approach is that the deaf person is not required to wear an uncomfortable device. Additionally, facial expressions can be included. Useful data can be easily obtained such as the depth and the color of images [2]. However, acquiring information related to the hand is more difficult when using the vision-based approach. Complex image processing is needed [4], and hand shape recognition is affected by the background condition. In addition, the lighting sensitivity may influence the accuracy of finger tracking [5,6]. Processes are performed using a computer, and the user always needs the camera, which makes it unusable for people with hearing impairment to use in their daily lives [17].

### 5.2. Challenges in SLR Using Gloves

**Figure 28:**
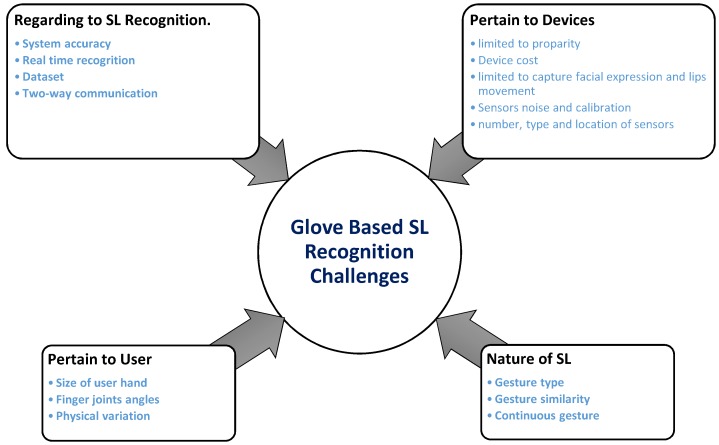
Categories of challenges for glove-based SL recognition.

A complex modelling framework is required to handle the features of the recognition system. After reviewing each study, we determined all the difficulties and problems that were addressed or encountered by previous researchers in SLR. The following are the most important obstacles classified into groups (Figure 28).

#### 5.2.1. Nature of SL

**Figure 29:**
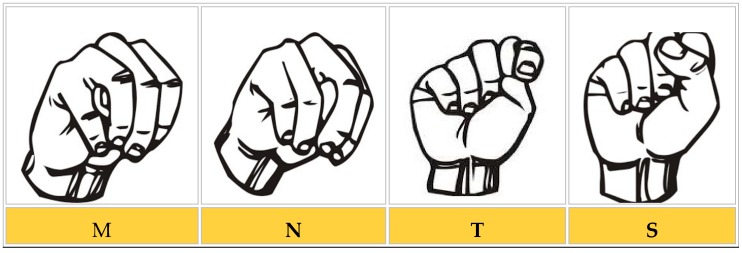
Similar postures in ASL, in terms of finger bending.

SL postures can either be static or dynamic. The classification of static hand postures is easier than that of dynamic hand postures, because movement history need not be considered [84]. Thus, most of the current recognition systems work well with static postures, because the error rates for dynamic postures are high due to insufficient training [18]. The similarity is another problem in SL; the movement or shape of a particular sign may be similar to another sign, so they may look similar in motion. For example, in the ASL alphabet, the letters “N”, “M”, “T”, and “S” are signed with a closed fist, as shown in Figure 29. At first glance, the posture of these five letters looks to be the same as [58]. This ambiguity may cause inaccurately classification, leading to low accuracy. The errors are mainly caused by letters equivalent to similar gestures such as V and U [59]. Continuous sign problem: In reality, signers perform signs in a continuous mode at a specified frequency and transition delay. Continuous gesture points denote sentences or phrases. The complexity of these points originates from a stream of signs being conducted that are not actually separated by discontinuity, such as speech [40].

There is an additional challenge regarding the meaningless movements between continuous gestures. Such transition periods between gestures are transitional frames known as Movement Epenthesis (ME). Many studies label these movements as a classification problem. They are not a universal system problem: different SLs exist worldwide, and each one has its own vocabulary and gestures. These languages are not familiar to people outside this certain community [56]. There is no universal system that provides an objective means of communicating for deaf people around the world [45,72].

#### 5.2.2. Pertain to User

In terms of the physical differentiation of each apprentice, the beginner cannot accurately synchronize his/her movement with that of a proficient person [40]. As a result, even if the beginner carries out the motion correctly according to his/her convictions, the listener may still have difficulties interpreting this motion; hence, the apprentice receives a low score because of the great variance from the skilled person [87]. Differences in several angular values of the finger joint should also be observed during gesture generation. These differences occur between different people, as well as within the same individual during perform gestures at different times [81]. Another issue is associated with signer dependency. A slight difference exists in the values, because the person cannot keep all his hands/fingers exactly the same [4]. One of the acquiesced facts is the variations among people. One of these differences, which has an effect on the action of the glove, is a difference in the size of the user’s hand [84]. The sensors are placed on gloves made of leather or cloth with several sizes; hence, the inappropriate use of the glove might adversely affect the accuracy of the performance [14].

#### 5.2.3. Pertain to Devices

A number of glove-based systems are available on the market. However, such devices have very high prices, which range from 1000 to 20,000 US dollars. The largest proportion of these groups of people with disabilities belongs to the low level of the economic pyramid, in which individuals cannot even dream of owning such devices [10,41]. This high cost diminishes the fact that it is a key tool in assisting people with disabilities in practice [42]. Limited Portability: The sentences and words of SL comprise many signs or gestures. Furthermore, most of these gestures are complex and need a computer for the purpose of recognition [11]. Thus, it is still difficult to move the SLR engine away from the computer [14,73]. Therefore, the SLR system is not portable and cannot be easily carried everywhere [56]. Most of SL alphabet is generated through the shape of the palm only [42]. However, many SL postures are formed by combining the shape of the hand, hand motion, and facial expressions in addition to the movement of the lips [36]. The glove can only record the movement of the hand or its shape and is not able to capture the rest of the important organs of the body to generate the gesture. Examples of this are arms, elbows, and the face. This may cause the inability to distinguish many movements such as ‘j’ and ‘z’, which are ignored because they involve moving gestures [43]. Glove measurement performance: In general, most publications provided a performance report on the sensors used in gloves. Information on the overall performance of the system in terms of accuracy and repeatability is rarely available [43]. No uniform methodology facilitates broad, independent comparisons of the recognition system. Society, as well as authors, will benefit from the development of standards that contribute to the advancement of the applied reality of such research [84]. Sensors noise: One of the most important factors is the selection of high-quality sensors to determine the accuracy of obtained data. However, when setting the goal of obtaining a low-cost product, less accurate sensors are used, which may generate noise in the acquired signal. This type of noise may affect the desired results, causing a loss of accuracy [62]. Calibration: According to the physical anatomy of people, there are differences in the size of a hand, finger extent, and thickness from one person to another [66]. Thereby, glove sensors could overlap with different finger positions for different users, which may influence glove performance. To reduce inexactitudes, most gloves need to be calibrated for a specific user [81,90]. Calibration is usually accomplished by requesting that the user put his/her hands in specified gestures (e.g., flat hand, flex the hand a few times, fist) [13,42]. Number of sensors: The real challenge of trajectory detection is to measure the common complementary function for SLR using a limited number of sensors [77,78]. However, if the number of sensors used is reduced, it will cause a loss of valuable data from the hand. This will lead to a loss of accuracy in translating the sign [6]. An increasing number of sensors in a particular design may raise the processor burden [84]. Type of sensors: Many types of sensors are available today. A sensor selection process to construct the interpreter glove has a positive or negative impact on the accuracy of the result [46,67]. For instance, several sensors are used to detect the bending of the finger. Each of these sensors has a certain way of working to determine the amount of finger curvature. However, not all of these ways are considered effective means for determining the bend [49]. Location of sensor: SL depends on the hand movement, which is composed of finger bend, wrist orientation, and the hand motion in free space. Accordingly, determining the right place for the appropriate sensor plays an important role in recognizing the largest number of SL gestures [42].

#### 5.2.4. Regarding SL Recognition

System accuracy: The beginning SLR systems suffered from poor accuracy; thus, to rip off this problem, the partial translation of the small movements was followed [39]. Furthermore, the existing glove face had difficulty obtaining complete body motions with great accuracy [42]. Real-time recognition: Over the years, gesture recognition has been studied. However, there are still numerous dilemmas with the capability to implement real-time recognition [78]. One of the challenges related to the glove is to obtain as good precision as possible when implementing fast movements to simulate real-time SL conversation [45]. Also, human hand has multiple degrees of freedom that provide a large number of possible movements, making it very difficult for modelling real-time system [81]. Even though modern CPUs have become increasingly faster, it may be difficult to process all incoming information with them at same time. Dataset: The availability of data is a point that is often overlooked but equally important in SLR; very limited datasets are available to the public [8]. Thus, the availability of reliable and accessible data has an important impact on helping researchers conduct hassle-free data collection. The benefit of using pre-existing data is to save time and effort and to be accurate when relying on reliable data [63]. Two-way communication: At present, the electronics market is provided with various SLR devices to support the abled individuals. However, few of these tools support two-way communication. Furthermore, most SLR tools are designed to teach SL and are not suitable for communication in real life [8]. The simulation program contains electronic circuits, all kinds of sensors, and other tools needed for glove design, and is very useful for the researcher, which speeds up the process of model building and avoids errors. Unfortunately, there is a limited number of such programs besides it lacks most of the basic components such as flex sensor, pressure sensor, contact pad, etc., which are needed to design the model and simulate the work correctly and accurately [48].

### 5.3. Recommendations

**Figure 30:**
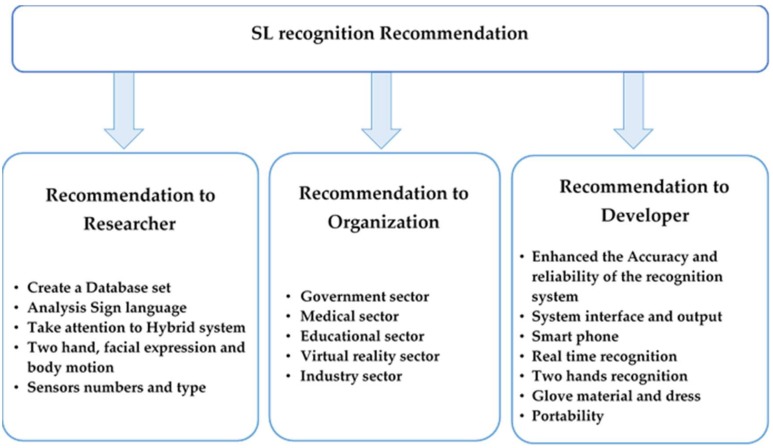
Categories of recommendations for SL recognition using gloves.

Helping people who suffer from weakness or loss of hearing and speech is the foremost incentive for providing a device that helps reduce the communication gap between them and other people. Accordingly, the following are the most important recommendations made by researchers (Figure 30).

#### 5.3.1. Recommendations to Developers

System cost: Cost is one of the challenges in developing the sign language (SL) discrimination system [12]. The vast majority of people who cannot hear or speak have low income or are below the poverty level [79]. Therefore, developing a cheap sign language recognition (SLR) system is the most important objective [38,59,85]. Accuracy and reliability: The desired outcome of developing a translation system is an effective, competent tool that provides support to people who need such a device to overcome the difficulty and barriers in communicating with others [52]. Accordingly, any system that has the capability to interpret SL is a fundamental means to eliminate these problems. Guaranteed accuracy and reliability is essential [10,36,64]. System performance can be improved by minimizing errors by using high-quality sensors; for instance, flex sensors, a triple axis ACC, and a gyroscope [7]. System interface and output: With regard to the output, a specking engine is added to translate the postures into speaking words, as well as text. The sound must be loud and clear enough to help the speaker [12,43,55,65]. To ensure device usability, a simple management interface is expected to be designed to simplify the handling of the device. Such an interface may help the signer to select and verify the proper expression interpretation [53,70]. With regard to the graphical display, the endeavor was undertaken to improve the performance of the device to make it an assistance tool that meets the purpose for which it was manufactured. Accordingly, it turned the developer’s attention towards translating speech/text into the animated sign language and three-dimensional models [5,42,65]. Smart phones: Modern developments in smart phones in terms of touch screen, beautiful graphical interface, smart applications, multiple wireless, and other specifications have made these devices indispensable for the vast majority of people [4,70]. All of these features made the phone an effective and ergonomic part of the SLR solution [7,11,76]. Real-time recognition: Providing a real-time response to a specific task in a recognition system is important in presenting instantaneous feedback to the user. Consequently, tracking and identification need to be manipulated as fast as possible [51,79]. Two-hand recognition: Most SL alphabets and postures can be performed with one hand [71]. However, a number of postures rely on the movement of two hands. The recognition system is equipped with two gloves to expand the system capacity and cover as many signs as possible [2,17,57]. Glove material and special dress: The recognition system incorporates an assortment of sensors mounted on a glove. The glove may be worn to carry out gesticulation for long durations and on a daily basis. Thus, the material used to make the glove needs to be elastic and comfortable so as not to restrict the speaker’s freedom to move. In order to protect the electronic circuits, it is desirable to add some protective layers to make the glove 100% waterproof [12,44]. In addition, the size of the designed board and the parts that are placed on the user’s body must be small to enhance the glove’s appearance (to be suitable for daily wear and does not cause embarrassment to the speaker [76]). The design of special clothing such as a whole jacket with the necessary number of sensors at fitting positions would be capable of pronouncing the postures and motions of dumb people [4,5,43]. Calibration: A good calibration would provide a precise synchronization of the actual hand movement and the virtual hand model. Thus, the exact angle value of the fingers can be obtained. The reason is that the glove consistently emits the values of an angle for the finger or hand [42,81]. Portability: The development of the glove-based translation system aims to assist persons with hearing disabilities in their daily lives. Therefore, the device must be portable to help the speaker take advantage of the solution’s features without connecting it to a computer [4,7,35,56,64,70,73,75].

#### 5.3.2. Recommendations to Organizations

The technique of recognizing gestures by using gloves is a new field of research that can be used in numerous applications of life. The different technologies available nowadays make it possible to address the recognition task from many perspectives [78]. Here are some of the most important sectors that will benefit from glove technology: Government sector: A gesture recognition device could enable the government to provide SL translation in public places, such as railway stations, airports, bank counters, hospitals, and hotels, where communication is observed among various individuals [21]. Medical sector: A gesture recognition product can be utilized for technical dexterity for training in several medical trades, such as surgery, because it simulates hand motions, thereby improving capability and accuracy [21,42]. Industry sector: A gesture recognition technology can be utilized in factories to perform multiple functions such as machine maintenance [4,43,82]. Virtual reality sector: The sensory glove can also be used in interactive applications of virtual reality technology, such as a wearable mouse glove, wearable keyboard glove, virtual musical appliances, computer games, simulated environment, etc. [27]. Educational sector: With the support of numerous education organizations, the glove device may be utilized as an educational tool, as it will assist younger children in learning the structure of a sentence, prepositions, and other grammatical characteristics that are difficult for a disabled person to handle [45].

#### 5.3.3. Recommendations to Researchers

Database set: The number of signs and gestures, as well as samples, for the database set of the recognition system should be increased on the basis of recommendations agreed upon by a large proportion of researchers. This should be considered in their future work to improve the performance of the recognition system [14,21,36,51,68,69]. The database set that belongs to many researchers in this area is very limited and may only contain numbers, alphabets, or a very limited number of the words [6]. Gestures are selected by considering the capability to correctly verify proficiency. The creation of a dictionary ensures that the database of gestures covers and involves the full space of all probable centers of SL expressions [76]. SL Analysis: SL has its own syntax rules and grammar to form sentences and phrases that consist of a series of gestures, as in spoken language [77,84]. These rules need to be considered when developing an SL translation system. The enforcement of strict grammar and context simplifies the SLR process [4]. In addition, the target spoken language rules must also be followed [43,65]. It must be taken into account that each community has local sets of SL; henceforth, it is necessary to create a standard universal SL across the world [8]. Furthermore, a global glove should be developed that would interpret such sign language [56]. Hybrid system: Concerning SLR, the sensor glove-based and vision-based approaches are essential. Each of these approaches has advantages and limitations; thus, designing an architecture depends on the suitable combination of these two approaches to produce an aspire system [54,76,78,82,84]. Two hands, facial expression, and body motion: As supported by several studies, which scanned data from a specified number of fingers, for system improvement and glove development, readings of all fingers need to be captured to increase the value of collected data [21]. Additionally, appending a pressure sensor to the middle and index fingers avoids misspelling caused by similar signs of letters. Moreover, adding a new filter to the character recognition program enables the full use of this sensor to discriminate between letters ‘U’ and ‘V’ [80]. Dynamic posture and arm gesture problems can be resolved by incorporating the investigation of arm joint angles by placing sensors on the arm [43,72]. Sensors on the elbow and possibly shoulder will be needed. Thus, the features of most sign language gestures would be recognized. It would also be very useful to obtain fusion information from two-hands parameters concerning sign language recognition, as well as facial expressions, head movement, and body posture [54,62,74]. Threshold value: Adding the maximum and minimum values of the threshold is useful for bypassing false inputs and errors in communication [45,78]. Numbers and types of sensors: Several substantial factors, such as using more sensors with higher quality, must be considered to fully grasp the meanings of real SL gestures, identify many SL gestures, improve system performance, and reduce errors [7,43,47]. Furthermore, the postures require different hand movements, such as wrist orientation, hand motion, or facial expressions. Therefore, data from various channels needs to be fused into the system [77]. A combination of different types of abduction sensors such as flex sensors, contact sensors, a gyroscope, and an ACC can capture more complicated gestures, as well as differentiate between similar letters to obtain a reliable, robust, and accurate recognition system [3,9,17,39,49,50]. However, using lightweight and fewer sensors helps to simplify the hardware complexity [8].

## 6. Important Issues in Previous Work

The researchers differed in their interests in the field of SLR using gloves. Several researchers focused on discovering an appropriate technique to capture the movement of fingers and palms, whereas others were interested in developing a recognition engine with good accuracy. In addition, SL analysis focused on several aspects, including gesture type, conversation style, and other issues. The important information needed by researchers who are interested in this field has been extracted to facilitate research. Table 3 provides a clear and simplified picture of the key points in the development of an SLR system through 62 development work. In terms of the type of sensor used, (17/62) studies were concerned with finger-bending measurement using bend detection sensors. Only (2/62) studies used inertial measurement unit sensors to record the motion of fingers and hands. Most of the studies (41/62) used both types of sensors to capture finger and hand movements. As for gesture type, the greatest focus was directed to the identification of static signs (45/62), whereas only (12/62) studies attempted to recognize static and dynamic gestures. Forty-seven of the 62 studies in literature were restricted to recognising isolated words, and (7/62) studies provided mechanisms for splitting continuous signs into separate signs to recognize them. The studies that developed a real-time signal recognition system numbered (32/62). The number of articles based on the analysis of SL in the recognition process was (12/62). The least number of studies (3/62) aimed to develop an SL translation system that could translate SL into speech and vice versa, i.e., translate speech into SL. However, most of the studies (47/62) developed a sign translation system into voice or text. A total of (27/62) scholarly studies considered the low cost of developing an interpreting system.

## 7. Patents

We conducted the search for a patent that presented regarding SLR using the sensory glove in google patents database. In 2007, the invention of Jose Hernandez-Rebollar is represented with No. US 2010/0023314 A1, called “ASL Glove with 3-Axis Accelerometers” in the United States Patent [92]. The patent is presented with 3-axis ACC glove apparatus to translating a dynamic and static gestures of ASL into text or speech. The apparatus consists of 3-axis ACC one per fingers, two on the back of the palm, two sensors on the upper arm to capture elevation and rotation of arm, and an angle sensor to measure elbow flexing and micro controller. The processed data were compared with trained gestures stored in a library to generate voice or written text as output. The claim of this work is that the device could detect motion, and position of the upper arm with respect to the shoulder. In 2011, the invention of Juan Álvarez Álvarez and Salvador León Gil is represented with No. 201130193, called “System and method of sign language interpretation” in the Spanish Patent [93]. The patent is presented a bidirectional communication interpretation system of sign language for a hearing-impaired individual. The system based on gloves provided with flexion sensors and one ACC was adapted to gather data on the hand position and movements. The microcontroller sends the processed sensors data via Bluetooth module to the mobile device to display 3D animation message and playback a voice message that corresponding to the recognized gesture and vice versa. In 2014, the invention of H.S. Shin and J.H. Park is represented with No. US20140028538A1, called “Finger motion recognition glove using conductive materials and method thereof” in the United States Patent [94]. The patent is presented with conductive materials configured to recognize the finger bend by applying a characteristic in which the glove which is made of conductive fibers. The glove equipped with pairs of contacts is placed on glove where knuckles of fingers are bent. A pair of contacts is positioned on the first surface of each finger. In 2015, the invention of Bosch (Shanghai) Smart Life Technology Ltd. is represented with No. US20170263154A1, called “Glove for Use in Collecting Data for Sign Language Recognition” in the United States Patent [95]. The patent is presented a glove equipped with multiple azimuth sensor fixed on locations corresponding to phalanges and metacarpal bones. The solution presented fewer sensors than were required to collected SL data. Claim 1 is that the azimuth sensors are only offset by phalanxes bones of the human hand on the gloves for the following fingers: index, a middle, ring, and little. Claim 2 of this patent is configured by a height sensor to sense data to measure hands’ heights; the plurality of height sensors is disposed of on the pair of gloves. In 2016, the invention of H. Bavunoglu and E.S. Bavunoglu, is represented with No. US 2016/0284236A1, called “System of converting hand and finger movements into text and audio,” in the United States Patent [96]. The invention is related to an SL recognition system that captures the motion achieved by the human hand and translatesit to written word and voice via a pair of gloves. Each glove is comprised of two flex sensors per finger to detect joint movements, four touch sensors between the fingers, and six 9 DoF IMU sensors (3 axis ACC, 3 axis gyroscope and 3 axis magnetometer). The microcontroller converts the raw data received from sensors through recognition algorithm to text and voice. In 2017, the invention of Motahar Sepehr is represented with No. WO2017208147A1, called “The intelligent system of digital glove for producing the words and sounds” in the international application and published under the patent cooperation treaty [97]. The lightweight intelligent glove was developed to produce the words and voice corresponding to SL gestures. The glove was equipped with the sensors and electronics circuit for making wireless signals. These signals are received by the user’s mobile phone, and one android application has been written for this invention. The specific application of this device is installed in the user’s mobile phone. The mentioned application changes the received information on the human speech of one of the languages such as English, Persian, Dutch, French, Italian, or other languages that are chosen by the user. In 2017, the invention of M. Mohandes and M.A. Deriche is represented with No. US 9,672.418 B2, called “Arabic sign language recognition using multi-sensor data fusion” in the United States Patent [98]. The patent is comprised of presented systems and methods for ArSL recognition. The recognition system includes circuitry to detect and track hand location and finger shape via 3-dimensional (3D) sensor data associated with one detected and tracked hand and one detected and tracked finger, wherein ten samples for each letter of 28 ArSLalphabet and for each LMC extracted feature are associated with respect to x, y, and z-axis of the 3D interactive space using pitch, roll, and yaw of hand. The LMC features were fed into a Linear Discriminant Analysis (LDA) classifier.

## 8. Conclusions

Developing an automatic machine-based SL translation system that transforms SL into speech and text or vice versa is particularly helpful in improving intercommunication. Progress in pattern recognition offers the promise of automatic translation systems, but many difficult problems need to be solved before they become a reality. Several aspects related to SLR technology, particularly SLR that uses a glove sensor approach, have been explored and investigated. The last set of included articles in the literature has been taxonomized into four main categories—framework, review and survey, development, and other hand gestures—based on the type of study. Likewise, the framework and development categories are divided into sub-categories. An in-depth analysis of literature assists in addressing and describing the challenges, benefits, and recommendations related to SLR using glove-based systems. The results reveal the available glove types, the sensors used for capturing data, the techniques that were adopted for recognition purposes, the identification of the dataset in each article, and the specification of the processing unit and output devices of the recognition systems. Many recommendations have been suggested by researchers to solve existing and anticipated challenges that provide many opportunities for research in this area. We hope that researchers will continue to adopt new technologies to establish a realistic system that may help people with hearing and speech disabilities improve their ability to integrate into society and reduce the communication gap. The major advantage of a sensory-based approach is that gloves can acquire data directly (degree of bend, wrist orientation, hand motion, etc.) in terms of voltage values of the computing device, thus eliminating the need to process raw data into meaningful values. Furthermore, this approach is not subject to environmental influences, for example, the location of the individual or the background conditions and lighting effects; thus, generated data is accurate. However, glove-based gesture recognition requires that the user wear a cumbersome data glove to capture hand and finger movements. This hinders the convenience and naturalness of human–computer interaction. The limitation faced by this approach is the inability to obtain meaningful data complementary to gestures to give the full meaning of the conversation, such as facial expressions, eye movements, and lip-perusing. In future research, weight and size must be considered when implementing such circuits to make a wearable, standalone system that is applicable to daily life without any interaction with a PC. Furthermore, efforts should be made to enhance the robustness of the system to enable effortless customization and extend the current methods to other types of applications, for example, to gesture-based mobile interfaces. Furthermore, sign languages have certain rules and a certain grammar for their sentence formation. These rules must be taken into account when translating a sign language into a spoken language. Additionally, it would be useful to develop a translation system capable of interpreting different sign languages. Finally, reliable segmentation methods should be developed to assist in continuous gestures recognition.

## Figures and Tables

**Table 1:** The most important details with regard to training datasets used in previous work.

Author	Device/Components	Language	Gesture	Samples per Gesture	Gesture Performer	Sample Size
[37]	five flex sensors	American Sign Language	four gestures			
[47]	five flex sensors, accelerometer, and tactile (contact) sensor	American Sign Language	set of 8 gestures A-H	10 times		80 samples
[69]	fiveflex sensors and ADXL335 accelerometer	American Sign Language	26 gestures alphabet and 10 more gestures to numbers			256 samples
[39]	8 touch sensors	American Sign Language	numbers 0 to 9 and the 26 English alphabets, A to Z	30 times		1080 samples
[64]	five flex sensors and a 3D accelerometer	American Sign Language	American National Corpus is used A-Z and “space” plus “full stop”	5 times	6 females and 4 males age between 20–26	1400 samples
[46]	six inertial measurement units (IMUs) accelerometer	American Sign Language	American Sign Language (ASL) letters without letters J and Z	one time	data was collected from 9 participants	216 samples
[70]	5DT Glove	American Sign Language	26 letters of the alphabet	3 times	three subjects familiar with the sign language	234 samples
[50]	five flex sensors, MEMS accelerometer (ADXL345), and contact sensor	American Sign Language	A-Z letters	10 times		
[71]	CybergloveTM	American Sign Language	50 ASL word	12 times	multiple person trained	120 samples
[80]	five fabric contact sensors, five flex sensors, and 3D accelerometer	American Sign Language	A to Z and “THE QUICK BROWN FOX JUMPS OVER THE LAZY DOG” statement	5 times	seven subjects, including six hearing and speech-impaired high school students and teachers	
[74]	Cyberglove	American Sign Language	74 distinct sentences from 107-sign vocabulary	2–6 times	eight signers	2393 sentences and 10,852 sign instances
[61]	two CyberGloves	Arabic Sign Language	100 two-handed signs	20 times	adult volunteer from the deaf community	2000 samples
[40]	DG5-VHand data gloves	Arabic Sign Language	40 sentences using an 80-word lexicon	10 times	24-year-old right-handed female	800 samples
[38]	flex and contact sensors	Australian Sign Language	120 static gestures	100 times		3600 samples.
[51]	flex sensors with 9-axis IMU sensor	Chinese Sign Language	Chinese phonetic alphabet including a, b, c, zh, and ch	30 times	two different individuals	150 samples
[78]	three-axis accelerometer (ACC) and multichannel electromyography (EMG)	Chinese Sign Language	72 signs	12 times	Two subjects: male (age 27) and female (age 25)	
[52]	9-axis accelerometer	English Alphabet	26 English alphabet	one time	one person	26 samples
[36]	Hall Effect sensor and accelerometer (ADXL-535).	English Numbers	English Numbers 0–9	20 times		200 samples
[77]	3-axis accelerometers (ACC) and electromyogram (EMG)	German Sign Language	seven words	10 times	eight subjects (6 males and 2 females, aged 27 to 41)	560 samples
[83]	EMG and 3-D Accelerometer	Greek Sign Language	60-word lexicon	10 times	three native signers	1800 samples
[79]	Three-flex sensors and three axes accelerometer	Indian Sign Language	four words namely HELLO, YES, SORRY, and PLEASE			
[55]	flex sensors and accelerometer	Indian Sign Language	eight commonly used words			
[75]	five flexure sensors and three accelerometers	Malay Sign Language	25 Bahasa Isyarat Malaysia (BIM) sign words are used	20 times	only one signer is used for creating signer	500 samples
[6]	10 tilt sensors and 3-axis accelerometer	Malaysian Sign Language	A, B, and C. 1, 2, and 3 ‘Saya’, ‘Makan’, and ‘Apa’.	10 times	three individuals	270 samples
[56]	five flex sensors and 3-axis accelerometer	Pakistani Sign Language	10 static gestures		(15 females and 15 males) who varied from13 to 45 years old	
[78]	5DT Data Glove	Spanish Alphabet	six movements	10 times		60 cases and 37 attributes
[49]	10 flex sensors attached to each finger and three-axis accelerometer	Taiwanese Sign Language	five words, namely, Lonely, Promote, Assist, Love, and Protect	each with 50 tests	five subjects	1250 tests
[35]	10 flex sensors and one accelerometer ADXL345	Vietnamese Sign Language	29 letters	50 tested for each letter		1450 samples
[4]	five ADXL202 accelerometers	Vietnamese Sign Language	23 Vietnamese-based letters with two postures for “space” and “punctuation	40 times	five different persons	200 samples

**Table 2:** The metric information of the articles in the previous study.

Article Ref.	Publisher	Article Type	Journal Name/Conference Name	Impact Factor/ Conf. Location	Citation	Year
[37]	IEEE	Journal	IEEE Transactions on Systems, Man, and Cybernetics, Part C (Applications and Reviews)	2.171	475	2008
[35]	IEEE	Journal	IEEE Transactions on Systems, Man, and Cybernetics	2.86	278	2011
[26]	Elsevier	Journal	Engineering Applications of Artificial Intelligence	3.74	109	2011
[58]	IEEE	Conference	Advances in Electronics, Computers, and Communications (ICAECC)	India	80	2007
[65]	IEEE	Conference	Automatic Face and Gesture Recognition	South Korea	57	2007
[56]	IEEE	Journal	IEEE Transactions on Instrumentation and Measurement	2.456	48	2013
[32]	Elsevier	Journal	Pattern Recognition	5.582	43	2014
[20]	IEEE	Conference	Information, Communications & Signal Processing	Singapore	33	2007
[28]	Elsevier	Journal	Engineering ApplicationsofArtificialIntelligence24	2.894	30	2010
[39]	IEEE	Conference	Body Sensor Networks (BSN)	USA	28	2011
[21]	IEEE	Journal	IEEE Transactions on Human–Machine Systems	2.493	27	2015
[29]	Elsevier	Journal	Neurocomputing	3.317	27	2007
[51]	IEEE	Journal	IEEE Multimedia	2.849	22	2008
[50]	IEEE	Conference	India Educators’ Conference (TIIEC)	India	19	2013
[52]	IEEE	Conference	Scientific Computing, Computer Arithmetic, and Validated Numeric	Germany	18	2007
[30]	Elsevier	Journal	Procedia Engineering	0.74	17	2012
[47]	IEEE	Conference	Sustainable Utilization and Development in Engineering and Technology (STUDENT)	Malaysia	17	2010
[38]	IEEE	Conference	Fourth International Conference on Technology for Education	India	15	2012
[44]	IEEE	Conference	Wearable and Implantable Body Sensor Networks (BSN)	UK	14	2012
[63]	IEEE	Conference	Global Humanitarian Technology Conference—South Asia Satellite (GHTC—SAS)	India	14	2014
[12]	IEEE	Conference	Global Humanitarian Technology Conference (GHTC)	USA	13	2016
[16]	IEEE	Conference	Advances in Electronics, Computers, and Communications (ICAECC)	India	13	2014
[54]	IEEE	Conference	Intelligent and Advanced Systems	Malaysia	13	2007
[3]	Elsevier	Journal	Procedia Computer Science	0.74	11	2015
[3]	Elsevier	Journal	Procedia Computer Science	0.74	11	2015
[55]	IEEE	Conference	Computer Engineering & Systems (ICCES)	Egypt	11	2013
[23]	IEEE	Conference	System of Systems Engineering	Singapore	10	2008
[25]	IEEE	Conference	Computing, Communications, and IT Applications Conference (ComComAp)	China	10	2013
[31]	Elsevier	Journal	Pattern Recognition	4.582	10	2008
[61]	IEEE	Conference	e-Technologies and Networks for Development (ICeND)	Lebanon	10	2014
[9]	IEEE	Conference	Electrical Engineering and Information Communication Technology (ICEEICT)	Bangladesh	8	2015
[45]	IEEE	Conference	Machine Learning and Cybernetics	China	8	2008
[57]	IEEE	Conference	Image and Vision Computing New Zealand	New Zealand	8	2009
[13]	IEEE	Conference	Systems Conference (SysCon)	USA	7	2014
[19]	IEEE	Conference	Global Humanitarian Technology Conference (GHTC)	India	7	2014
[24]	IEEE	Journal	The Computer Journal	0.711	7	2010
[2]	IEEE	Conference	Human Computer Interactions (ICHCI)	India	6	2013
[11]	IEEE	Journal	IEEE Sensors Journal	2.512	6	2016
[1]	IEEE	Conference	International Conference on Control, Automation, and Systems	South Korea	5	2015
[6]	IEEE	Journal	International Journal of Computer Applications	0.26	5	2015
[8]	IEEE	Conference	International Conference on Electronic Measurement & Instruments	China	5	2015
[10]	IEEE	conference	Electron Devices and Solid-State Circuits (EDSSC)	China	5	2015
[64]	IEEE	Conference	Innovative Computing, Information, and Control	Japan	5	2007
[4]	IEEE	Conference	Circuits and Systems (MWSCAS)	USA	4	2015
[5]	IEEE	Conference	Multi-Topic Conference (INMIC)	Pakistan	4	2014
[18]	Elsevier	Journal	Procedia Engineering	0.74	4	2012
[40]	IEEE	Conference	Humanitarian Technology Conference—(IHTC)	Canada	4	2014
[7]	IEEE	Conference	Computing for Sustainable Global Development (INDIACom)	India	3	2015
[14]	IEEE	Conference	Computational Science and Technology (ICCST)	Malaysia	3	2016
[15]	IEEE	Journal	IEEE Sensors Journal	2.512	3	2016
[27]	Elsevier	Journal	Procedia Computer Science	0.74	3	2014
[33]	Elsevier	Journal	Pattern Recognition Letters	1.995	3	2017
[59]	IEEE	Journal	IEEE Transactions on Biomedical Engineering	3.577	3	2016
[60]	IEEE	Conference	Computer & Information Technology (GSCIT)	Tunisia	3	2015
[17]	IEEE	Conference	Control, Decision, and Information Technologies (CoDIT)	St. Julian’s, Malta	2	2016
[34]	IEEE	Conference	Contemporary Computing (IC3)	India	2	2015
[66]	IEEE	Conference	Communication Systems and Network Technologies (CSNT)	India	2	2015
[67]	IEEE	Conference	Technology Management and Emerging Technologies (ISTMET)	Indonesia	2	2014
[22]	IEEE	Conference	Computational Science and Computational Intelligence (CSCI)	USA	1	2016
[43]	IEEE	Conference	Electronic Devices, Systems, and Applications (ICEDSA)	United Arab Emirates	1	2016
[46]	IEEE	Conference	Ecuador Technical Chapters Meeting (ETCM)	Ecuador	1	2016
[49]	IEEE	Conference	Electrical Engineering, Computing Science, and Automatic Control (CCE)	Mexico	1	2014
[36]	IEEE	Conference	Radio Science Conference (NRSC)	Egypt	0	2017
[41]	IEEE	Conference	Michael Faraday IET International Summit 2015	India	0	2015
[42]	IEEE	Conference	Automatic Control and Dynamic Optimization Techniques (ICACDOT)	India	0	2016
[48]	IEEE	Conference	Circuits and Systems (MWSCAS)	United Arab Emirates	0	2016
[53]	IEEE	Conference	India Conference (INDICON)	India	0	2015
[62]	IEEE	Conference	Information Science, Signal Processing, and their Applications (ISSPA)	Canada	0	2012

**Table 3:** Summary of the most important issues in previous work.

Ref.	Sensor Used for			Gesture		Data Set				SignType		Execute Real Time		No. of Hands		Interfaced				Design Hardware Module	Software Application	Language Analysis	Communication		Low Cost System	Mobility/Portable	Use Start/Stop Signs
	Bend Detection	Move Detection		Static	Dynamic	Number	Alphabet	Word/Phrases	Few Gesture	Isolated	Continuous			One Hand	TwoHand	PC	LCD/Speake	Mobile	3DAnimation				One way	Two ways			
[21]	*			*					*	*				*		*				*	*	*	*		*		
[2]	*			*						*			*	*			*			*			*		*	*	
[7]	*			*			*			*			*	*			*			*			*			*	
[44]	*			*					*	*				*		*				*			*		*		
[12]	*			*				*		*			*	*			*			*			*		*	*	
[37]	*			*					*	*			*	*			*			*			*		*	*	
[68]	*			*			*			*				*			*						*		*		
[65]	*			*			*			*			*	*			*			*			*		*	*	
[39]	*			*		*	*			*			*	*			*			*			*		*	*	
[36]	*			*						*			*	*			*			*			*		*	*	*
[80]	*		*	*	*			*		*				*						*							*
[4]	*		*	*			*			*			*	*				*		*			*			*	
[59]	*		*	*			*			*			*	*			*			*		*	*			*	
[3]	*		*	*						*					*	*				*			*		*		
[58]	*		*	*			*			*				*		*				*			*			*	
[50]	*		*	*	*		*			*			*	*			*			*			*		*	*	
[47]	*		*	*					*	*				*		*	*			*			*		*		
[8]	*		*	*				*		*			*	*		*		*		*				*			*
[64]	*		*	*	*		*			*			*	*		*							*				*
[10]	*		*	*		*				*				*		*				*			*		*		
[51]	*		*	*		*	*			*			*	*			*			*			*		*	*	*
[54]	*		*	*	*			*		*			*	*		*			*			*	*				
[81]	*		*	*				*			*		*	*		*			*	*	*		*				
[43]	*		*	*			*			*				*													*
[71]	*		*	*	*			*		*			*	*		*						*	*				
[74]	*		*	*	*			*			*			*		*					*	*	*				
[61]	*		*	*			*			*				*		*						*	*				
[70]	*		*	*			*			*			*	*		*			*		*		*				
[76]	*		*	*	*									*		*				*	*		*				
[53]	*		*					*		*				*		*				*		*	*			*	*
[52]			*	*			*			*				*		*		*				*	*				
[42]	*		*	*		*	*			*				*		*									*	*	
[39]	*			*			*			*			*	*			*			*			*		*	*	
[86]			*	*			*			*			*	*				*		*	*		*			*	
[57]	*			*			*			*			*	*			*			*	*		*		*	*	
[27]	*		*	*	*			*		*						*							*				*
[40]	*		*	*	*			*			*				*	*							*				
[49]	*		*	*						*				*		*				*		*	*		*		
[69]	*		*	*					*	*			*	*				*		*	*		*			*	
[73]	*		*	*				*		*			*		*	*							*				
[72]	*		*	*	*			*		*					*	*							*				
[41]	*			*					*	*				*		*				*		*	*		*		
[9]	*			*										*						*					*	*	
[38]	*			*				*		*					*		*						*		*		
[45]	*								*				*	*						*	*			*	*	*	
[24]	*		*	*			*			*				*		*				*		*	*		*		*
[66]	*		*	*					*	*			*	*			*			*			*		*	*	
[5]	*		*	*										*			*									*	
[35]	*		*	*			*			*				*		*				*	*		*				
[55]	*		*	*					*				*	*			*			*							
[67]	*		*										*		*			*		*			*			*	
[56]	*		*	*							*		*	*		*			*	*	*		*		*		
[6]	*		*	*					*				*	*			*			*			*		*	*	
[11]	*		*	*					*	*			*		*		*			*				*		*	
[87]	*		*	*															*								
[82]	*		*	*	*			*		*	*																*
[83]			*	*	*			*			*		*		*	*				*			*		*		
[42]	*		*	*				*		*			*		*			*				*	*			*	
[13]				*					*	*				*													
[91]	*									*						*											

Note: we use “*” to indicate the elements used in previous work.
